# Phenotypic Pattern-Based Assay for Dynamically Monitoring Host Cellular Responses to *Salmonella* Infections

**DOI:** 10.1371/journal.pone.0026544

**Published:** 2011-11-03

**Authors:** Xiaozhou Mou, Shuying Wan, Yifei Li, Shanshan Zhang, Mingjiao Sun, Fanglong Liu, Huiying Fu, Xue Zhang, Haiying Liu, Qian Cao, Yuehai Ke, Charlie Xiang

**Affiliations:** 1 State Key Laboratory for Diagnosis and Treatment of Infectious Diseases, The First Affiliated Hospital, Zhejiang University School of Medicine, Hangzhou, China; 2 Institute of Molecular Pathology and Program in Molecular Cell Biology, Zhejiang University School of Medicine, Hangzhou, China; 3 Molecular Diagnosis Division, Zhejiang-California International Nanosystems Institute (ZCNI), Hangzhou, China; 4 State Key Laboratory for Molecular Virology and Genetic Engineering, Institute of Pathogen Biology, Chinese Academy of Medical Sciences and Peking Union Medical College, Beijing, China; Indian Institute of Science, India

## Abstract

The interaction between mammalian host cells and bacteria is a dynamic process, and the underlying pathologic mechanisms are poorly characterized. Limited information describing the host-bacterial interaction is based mainly on studies using label-based endpoint assays that detect changes in cell behavior at a given time point, yielding incomplete information. In this paper, a novel, label-free, real-time cell-detection system based on electronic impedance sensor technology was adapted to dynamically monitor the entire process of intestinal epithelial cells response to *Salmonella* infection. Changes in cell morphology and attachment were quantitatively and continuously recorded following infection. The resulting impedance-based time-dependent cell response profiles (TCRPs) were compared to standard assays and showed good correlation and sensitivity. Biochemical assays further suggested that TCRPs were correlated with cytoskeleton-associated morphological dynamics, which can be largely attenuated by inhibitions of actin and microtubule polymerization. Collectively, our data indicate that cell-electrode impedance measurements not only provide a novel, real-time, label-free method for investigating bacterial infection but also help advance our understanding of host responses in a more physiological and continuous manner that is beyond the scope of current endpoint assays.

## Introduction

Interactions between pathogens and their hosts are complex and dynamic [1]. The outcomes of these interactions reflect the properties of the pathogenic agent and the ability of the host to respond to infection. A better understanding of the mechanisms underlying host-pathogen interactions should contribute to improvement in treatment and control of infections. Characterization of host-pathogen interactions has advanced greatly over the past two decades, mainly through employment of cell cultures and animal models [1], [2], [3]. However, improved understanding of the cellular mechanisms of such interactions remains elusive.

Although some methods have been developed to study the interaction between pathogens and hosts, most are static endpoint assays and use a single-time point after infection to assess the cellular response [4], [5]. Cell behaviors are normally studied by microscopic examination of cell density and morphology [4]. In addition, endpoint assays use expensive reagents such as antibodies or fluorescent probes and require tedious cell manipulation, making them unsuitable for high-throughput analysis and increasing the exposure of lab workers to pathogens.

Label-free cell detection using cell-electrode impedance readout was first introduced by Giaever and Keese almost in two decades ago [6], [7]. This detection is noninvasive to cells, allowing for continuously monitoring of cellular responses to stimulations in real time. A label-free detection system (xCELLigence system, Roche Applied Science and ACEA Biosciences Inc.) was developed recently based on microelectronic biosensor technology. Electrode impedance is primarily determined by the ion environment at the electrode/solution interface and in the bulk solution. The cells on the electrode act as isolation and alter the ionic environment at the electrode/solution interface, leading to an increase in electrode impedance. Therefore, cell impedance is mainly determined by the number of cells attached to the electrodes, while cell adhesion or spreading also leads to changes in cell impedance (http://www.aceabio.com). Recently, this system has been applied to a wide range of cell-based assays, including quality control of cell cultures [8], assays for cytotoxicity and proliferation [9], cell adhesion and spreading [10], receptor kinase activation [11], mast cell activation [12], and G protein-coupled receptor activation [13].

In this work, we developed an impedance-based assay for real-time monitoring of the cellular response to bacterial infection. We applied this method to studying the infection of intestinal epithelial cells by *Salmonella*. The resulting time-dependent cell response profiles (TCRPs) reflected the entire process of *Salmonella* infection. The specificity and sensitivity of the TCRPs of a *Salmonella* infection were confirmed using currently available endpoint assays. Our data indicate that the TCRP-based technique can provide continuous and quantitative information for studying the cellular mechanism of interactions between *Salmonella* or other bacteria with diverse host cells.

## Results

### Dynamic monitoring of the intestinal epithelial cells in response to *Salmonella* infection

**Figure 1:**
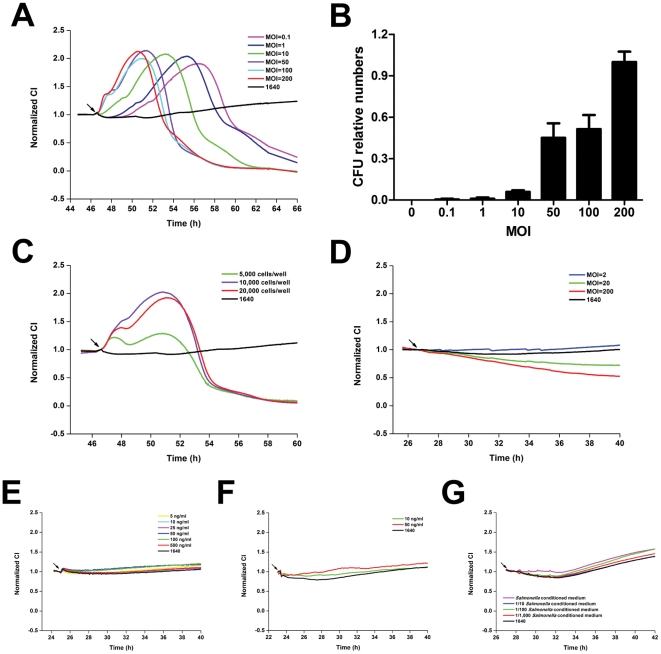
Dynamic monitoring of intestinal epithelial cell responses to *Salmonella* infection. (A) HT-29 cells (10,000 cells per well) were seeded into E-plates and infected with MOIs 0.1, 1, 10, 50, 100, or 200 of *S. typhimurium*. (B) Effect of increasing MOI on invasive bacterial count. The number of intracellular bacteria in cells infected with *S. typhimurium* at MOI of 200 was sets as 1.0. Results are mean values ± S.E.M from three independent experiments done in triplicate. (C) Initial inoculums of 5,000, 10,000, or 20,000 cells per well of HT-29 cells were seeded into E-plate wells. Fixed amounts (2×10^6^ cfu) of *S. typhimurium* were added. (D) TCRPs of HT-29 cells in response to *S. typhimurium* bacterial components or culture products. HT-29 cells (10,000 cells per well) were seeded into E-plates. The next day, cells were treated with heat-inactivated bacteria (D), LPS (E), flagellin (F) or conditioned medium of *S. typhimurium* (G) and CIs were continuously monitored every 5 min. Arrows in all figures represent time points after bacteria or stimulator addition. Representative curves are an average of four replicate wells.

Previously reports indicated that cell-electrode impedance signals reflect the changes in cell status in response to chemical compound treatments [10], [13], [14]. In this work, we adopted impedance-sensing technology to dynamically monitor intestinal cells in culture in response to *Salmonella typhimurium* infection. Figure 1A shows a TCRP produced by intestinal epithelial cells in response to different multiplicities of infection (MOIs) of *S. typhimurium*. The infection induced an immediate increase in cell Index (CI) following by a decline in CI values compared with the TCRP of uninfected cells. Moreover, a correlation was observed between the amount of bacteria used for infection and the increase in CI rate. With decreasing MOI from 200 to 0.1, the initial CI increase rate was gradually reduced and the delayed TCRPs were observed. For an MOI range from 0.1 to 200, the time to reach the CI peak was delayed in cells in lower MOI conditions, but the peak CI values were almost the same across the MOI range, demonstrating similar yet shifted CI curve profiles.

As intracellular bacteria, *Salmonella* invasion of non-phagocytic cells is a crucial step in pathogenesis. To test the effect of MOI on *S. typhimurium* invasion, HT-29 cells were infected with *S. typhimurium* at MOIs of 0, 0.1, 1, 10, 50, 100, or 200. Figure 1B shows that the number of intracellular bacteria increased with increasing MOI. To further investigate *S. typhimurium*-induced TCRPs in HT-29 cells, titrations of different numbers of cells were seeded into E-plate wells and infected with a fixed number of bacteria. Compared with TCRPs produced by infection with 10,000 or 20,000 cells, the response profiles generated using the lower number of 5,000 cells exhibited two distinctive peaks and lower CI peak amplitudes (Figure 1C). Cell number-dependent and bacteria number-dependent TCRPs are in Figure 1A and Figure 1C, and suggest that the observed TCRPs reflected the complex, dynamic bacteria infection process.

*S. enteritidis* is a closely related strain of *S. typhimurium* that causes nearly identical diseases in humans. To test whether these two closely related strains induced similar TCRPs in host cells, HT-29 cells infected with *S. enteritidis* were monitored using the xCELLigence system. As shown in Figure S1A–B, TCRPs induced by *S. enteritidis* infection were almost identical to TCRPs from *S. typhimurium*-infected cells (Figure 1A and Figure 1C), suggesting a similar host cell response to both *Salmonella* strains.

Conserved microbial products such as lipopolysaccharides (LPS) and flagellin trigger a variety of host responses in immune cells such as macrophage, dendritic cells and neutrophils, leading to the activation of NF-KB and MAPK and the production of pro-inflammatory cytokines [15], [16]. To test whether the observed TCRPs were induced by living bacteria or bacterial components, we treated HT-29 cells with bacterial components. As shown in Figure 1E–F, LPS and flagellin derived from *S. typhimurium* did not lead to CI changes. Furthermore, HT-29 cells were also insensitive to *S. typhimurium*-conditioned medium (Figure 1G, Supplemental Method S1). However, heat-inactivated bacteria induced a decrease in CIs (Figure 1D). Collectively, these findings indicated that TCRPs were induced only by living *Salmonella* bacteria but and by bacterial components.

### TCRPs predicting host cell physiological changes from *Salmonella* infection

**Figure 2:**
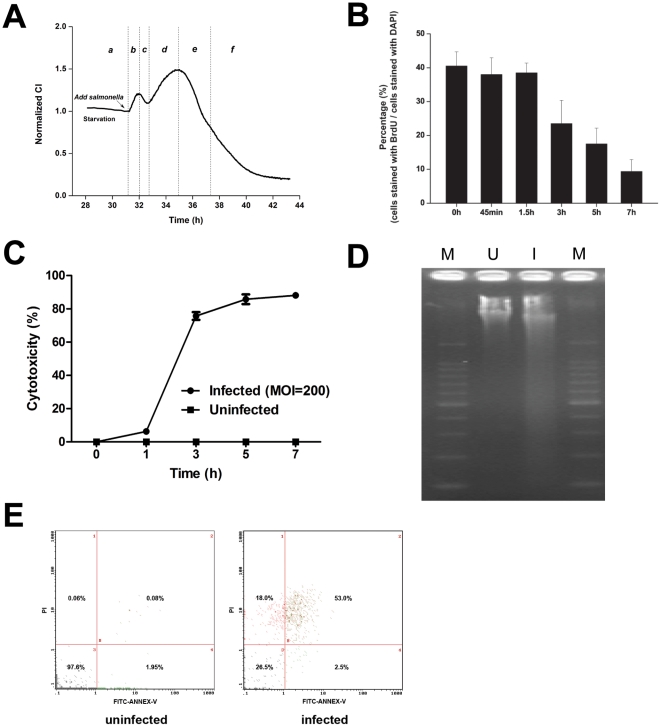
TCRP predicts host cell survival after *Salmonella* infection. (A) Schematic graph of the TCRP of HT-29 cells in response to *S. typhimurium* infection (MOI = 200). TCRP was divided into phases a through f along the time-axis. Cell samples for endpoint assays at each phase were collected and analyzed. Arrow represents the time points of bacterial addition. (B) Effects of *S. typhimurium* on cell proliferation. HT-29 cells were infected with *S. typhimurium* (MOI = 200) for 0, 0.75, 1.5, 3, 5 or 7 h and stained with BrdU and DAPI. Data are expressed as mean ± SD (n = 5). (C) Cytotoxic effect of *S. typhimurium* on HT-29 cells. Cells were infected with *S. typhimurium* at an MOI of 200 or left uninfected as a control. Cytotoxicity at 0, 1, 3, 5, 7 h was evaluated by LDH release assay (n = 4). (D, E) Cell death induced by *S. typhimurium* infection. HT-29 cells (10^6^ cells per well) were seeded into 6-well plates and infected with SL1344 at MOI = 200. Cell death induced by *Salmonella* was analyzed with DNA fragmentation assay (D) and flow cytometer method (E). I, DNA isolated from HT-29 cells infected *Salmonella* for 7 h; U, DNA isolated from uninfected cells at the same time point. M, DNA molecular marker (100 bp DNA ladder).

A unique two-peak TCRP could be detected by dynamic monitoring of the entire process of the cell response to *Salmonella* infection (Figure 1C and Figure 2A). To elucidate the cellular events underlying the two peaks, we performed BrdU incorporation assays on infected cells at different time points representing specific CI pattern features (Supplemental Method S1). BrdU is an analog of DNA precursor thymidine that can incorporate into cellular DNA during cell proliferation. As shown in Figure 2A, the host-*Salmonella* interaction process was classified into four phases of pre-infection (a), first peak (b and c), second peak (d and e) and CI steady decline (f). As shown in Figure 2B, BrdU incorporation was not affected during the first 1.5 h of infection (corresponding to phase b and c). In contrast, the BrdU incorporation rate steadily declined during the second peak and in the CI decline (phase d, e and f), indicating that the proliferation inhibition occurred immediately after the first TCRP peak.

To investigate the toxic effects of the *Salmonella* on epithelial cells, HT-29 cells were infected with *Salmonella* at an MOI of 200 and toxicity was indirectly measured by lactate dehydrogenase (LDH) release assay after 1, 3, 5, and 7 h of infection. LDH is a cytosolic enzyme and its release into the culture supernatant is an indicator of plasma membrane damage and cell death. As shown in Figure 2C, *Salmonella* induced a time-dependent release of LDH. However, the negative control (uninfected) cells exhibited no detectable LDH release.

DNA fragmentation has been used to measure the final steps of apoptosis. A very low level of spontaneous fragmentation was seen in DNA isolated from uninfected cells, while prominent fragmentation was evident in cells that had been infected for 7 h with *S. typhimurium* at an MOI of 200 (Figure 2D, Supplemental Method S1). To test whether infected cells underwent apoptosis, HT-29 cells collected at 7 h post-infection (corresponding to the beginning of the CI steady decline phase f) were analyzed by flow cytometry. As shown in Figure 2E, the viability of *Salmonella*-infected cells was three-quarters lower than the uninfected cells that served as controls, indicating that the infected cells were undergoing apoptosis. These findings suggested that the unique TCRP might provide important temporal information about cell physiological changes induced by *Salmonella* infection.

### TCRPs of *Salmonella* infection correlate with cytoskeleton-associated morphological changes

**Figure 3:**
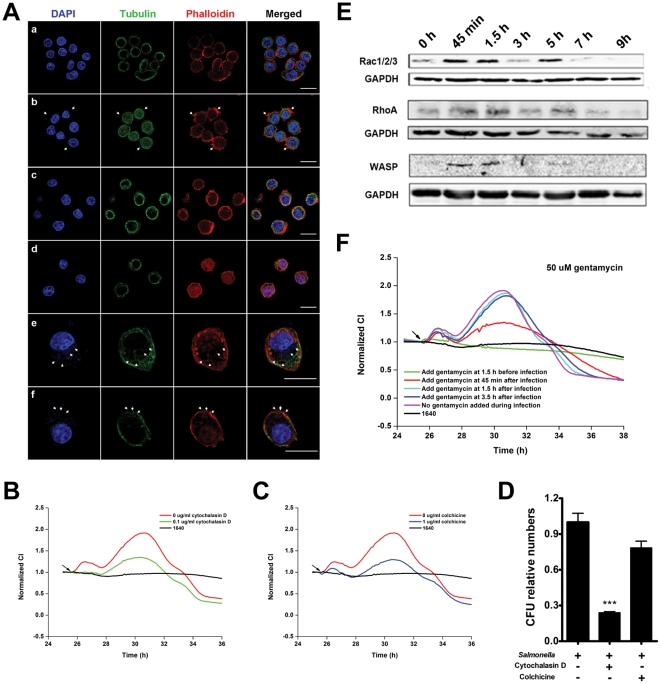
Correlation of *Salmonella* infection TCRP of intestinal cells and cytoskeleton-associated morphological dynamics. (A) Immunofluorescence images of HT-29 cells infected with *S. typhimurium*. (a) uninfected cells; (b) cells at 45 min post-infection. Actin-containing stress fibers are visible, as large accumulations of polymerized actin surrounding invading bacteria (arrows). (c and d) Cells at 1.5 h and 3 h post-infection. Actin cytoskeleton had normal architecture. (e) Cells at 5 h post-infection. Arrows, membrane ruffling and Salmonella-containing vacuoles (SCV). (f) Cells at 7 h post-infection. Arrows, membrane rupture and release of intracellular bacteria. (B) After pretreatment with cytochalasin D for 1.5 h, HT-29 cells were infected with *S. typhimurium* (MOI = 200). (C) After pretreatment with colchicine for 1.5 h, HT-29 cells were infected with *S. typhimurium* (MOI = 200). (D) HT-29 cells were pretreated with mock medium, cytochalasin D or colcocine for 1.5 h and infected with *S. typhimurium* at an MOI of 200. The number of invasive bacteria was determined by gentamycin protection assay. Data are mean ± S.E.M with the number of intracellular bacteria in mock-treated cells set as 1.0. ***, p<0.001 (*Student's t*-test). (E) Expression levels of cytoskeleton-associated proteins during *Salmonella* infection measured by western blotting. (F) Profiles of *Salmonella* infection after treatment of HT-29 cells with gentamycin (50 µg/ml) during infection. Arrows in B, C, F: bacterial addition points. Representative curves are an average of four replicate wells.

*Salmonella* species modulate the actin cytoskeleton of epithelial cells, resulting in bacterial entry followed by host cell morphological changes [1], [2], [4]. To address whether the TCRPs induced by *Salmonella* infection correlated with the host cell morphology, infected cells were collected at different phases according to Figure 2A and analyzed by immunofluorescent assay and scanning electron microscopy (Supplemental Method S1). As depicted in panel b of Figure 3A, a significant amount of filamentous actin accumulated in the proximal cell membrane. Pseudopodia extension and membrane ruffling were observed at 45 min after infection corresponding to phase b of the TCRP, suggesting that the first peak was indicative of *Salmonella* entry into cells (Figure 3A.b, and Figure S2.b). From 1.5 h to 3 h after infection (phase c, d of TCRP), the actin cluster (or accumulation) could not be detected (Figure 3A.c–d). This actin re-arrangement correlated with the rise of the second peak, as evidenced by the enlargement in cell shapes (phase e). Membrane ruffling and formation of *Salmonella*-containing vacuoles (SCVs) were visualized at 5 h after infection (Figure 3A.e, Figure S2.e). The size of infected cells was larger than uninfected cells, indicating that the second peak was associated with cellular enlargement induced by bacterial replication. At 7 h after infection, membrane rupture was seen at the site of intracellular bacterial release and cell shrinkage was observed (Figure 3A.f, Figure S2.f).

To further investigate the role of actin and microtubule structure in *Salmonella*-induced host cell morphologic dynamics, we used cytochalasin D, an inhibitor of actin filament polymerization, and colchicine, a microtubule depolymerizing agen. At a concentration that did not affect the CI, cytochalasin D completely inhibited the first peak and partially inhibited the second peak induced by *Salmonella* infection (Figure 3B). In addition, the number of invasive bacteria was significantly decreased after pretreatment of epithelial cells with cytochalasin D (Figure 3D). In cells pretreated with colchicine, both peaks were inhibited partially (Figure 3C). As shown in Figure 3D, the number of invasive bacteria was not significantly changed. Gentamycin is a membrane-impermeable antibiotic that kills bacteria only outside of cells [17]. As shown in Figure 3F, cells pretreated with gentamycin at a bactericidal concentration showed no impedance change compared to cells without *Salmonella* infection. Cells treated with gentamycin at 45 min post-infection showed a lower second peak. Treatment of cells with gentamycin at 1.5 h or later had no effect on the impedance profile. These findings indicated that the first peak corresponded to bacterial internalization, and the invasion process occurred before 1.5 h post-infection. The second peak was therefore mainly associated with the effects of *Salmonella* inside cells and was independent of bacteria outside of cells.

Effector proteins encoded by the SPI-I and SPI-II play important roles in *Salmonella*-induced endocytosis via regulating actin cytoskeletal changes by cycling between inactive GDP-bound and active GTP-bound states of the Rho GTPase subfamily of small G proteins [3], [18], [19]. Expression levels of Rac1, RhoA, and WASP were determined by Western blotting (Figure 3E). The expression levels increased in close association with the TCRP (Figure 2A) and actin cytoskeleton dynamics, as shown in Figure 3A. This indicated that the two impedance peaks might reflect cytoskeleton dynamics associated with different stages of the *Salmonella* infection process.

### Broad application of the cell-based infection model

**Figure 4:**
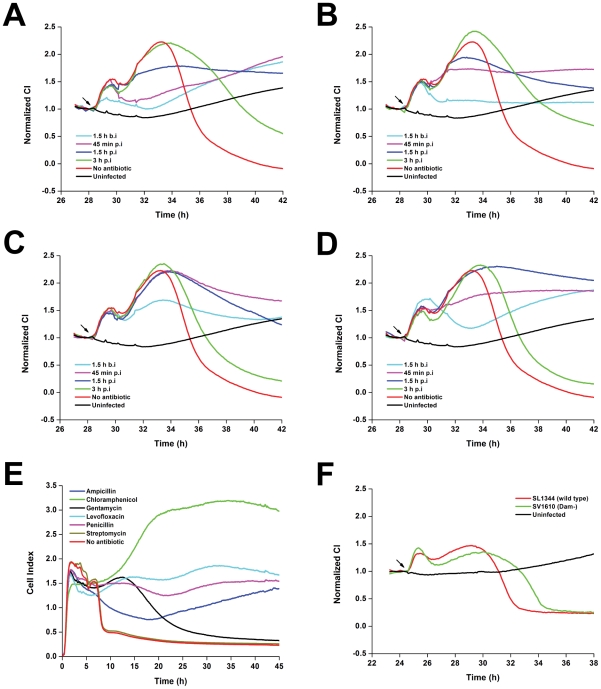
Broad application of cell-based infection model. (A–D) Dynamic monitoring of HT-29 cells during *S. typhimurium* infection after antibiotic addition at different time points. Kanamycin (A), ampicillin (B), chloromycetin (C) and levofloxacin (D). b.i, before infection; p.i, post-infection. (E) Intracellular activities of antibiotics against *Salmonella*. *S. typhimurium* strain SL1344 was phagocytosed by J774A.1 cells and extracellularly exposed to antibiotics at 10×MIC. (F) *S. typhimurium* Dam deficiency results in extended host cell survival. HT-29 cells were infected with *S. typhimurium* wild type stain SL1344 or Dam deficient stain SV1610 at MOI = 200. Arrows, bacterial addition. Representative curves are an average of four replicate wells.

As described above, treatment of cells with gentamycin affected the TCRPs of *Salmonella* infection. Different classes of antibiotic drugs inhibit bacteria by different mechanisms. To analyze the potential of the cell-based infection model for antibiotic studies, five typical antibiotics of different classes were added to the infection system at different time points. Gentamycin and kanamycin, which are in the class of aminoglycosides protein synthesis inhibitors (bactericidal), showed similar TCRPs (Figure 3F and Figure 4A). Treatment of cells with antibiotics at different time points affected the two peaks of the TCRPs. Furthermore, the earlier the antibiotics were added, the longer the cells survived. Cells treated with ampicillin and vancomycin, which work by inhibiting synthesis of the bacterial cell wall, showed different TCRPs. Different treatment time points did not affect the first peak. However, the earlier the antibiotics were added, the lower the decline in the second peak (Figure 4B, C). Levofloxacin is in the class of quinolone antibiotics, which have a broad spectrum of action. Treatment of cells with levofloxacin also lowered the peak amplitude (Figure 4D). As shown in Figure 4, adding these antibiotics before 3 h post-infection rescued the host cells from cell death. Infected cells treated with antibiotics at 3 h post-infection survived longer than cells without antibiotic treatment. These results indicated complex antibacterial mechanisms among different antibiotics that could be monitored with this cell-based assay.

Microarrays are currently widely used for studying cellular responses to bacterial infections [20], [21]. However, time points for cell collection are empirically determined and the phenotypes of host cells at these time points are usually unknown. As a dynamic method, we hypothesized that the cell-based infection model could help to select time points for sample collection (Supplemental Method S1). Using the profile in Figure 2A, time points of 0, 0.75, 3 and 7 h post infection were chosen, corresponding to the four infection phases. Expression of 27,155 (49.7% of all probe sets) transcripts was detected for at least one time point during *S. typhimurium* infection. A coefficient variation (CV) curve was used to find 272 differently expressed genes (Figure S3B, Table S1). Most genes showed increased expression in response to *S. typhimurium* infection (Table S1). The transcriptional program stimulated by *Salmonella* infection included several genes with pro-inflammatory products such as chemokines, cytokines and their receptors (CCL20, CCR7, CSF2, CSF3, CXCL1, CXCL2, CXCL3, IL8, IL11, IL24, IL6R, TNFRSF10B, TNFRSF9, and TNFRSF25). *S. typhimurium* also induced the expression of a number of transcription factors (ATF3, JUN, EGR1, and EGR4) that amplify the immune response. A gene ontology analysis indicated that genes involved in cell communication, response to external stimulus, receptor binding, cytokine activity, and chemotaxis were significantly overrepresented (Table S2). Pathway analysis demonstrated that genes involved in the Jak-STAT signaling pathway, ligand receptor interaction, the MAPK signaling pathway, and the ErbB signaling pathway were significantly enriched (Table S3).

The exposure of cells to pathogens that are deficient in a specific component or gene can identify components that are necessary for generating or altering the host response. Mutants of *S. typhimurium* lacking DNA adenine methylase are attenuated for virulence in vivo [22]. As shown in Figure 4F, cells infected with *S. typhimurium* SV1610 (Dam^−^) had profile similar to SL1344 (wild type). However, the TCRP of SV1610 infection showed a broad second peak, indicating that *Salmonella* lacking adenine methylase also had invasive capability, but resulted in delayed cell death during infection of epithelial cells.

## Discussion

Host-bacterial interactions are complex and dynamic processes. Understanding the mechanisms of such interactions helps prevent and control infectious diseases. Current technologies for assaying cellular responses to pathogenic infection typically analyze only one cellular function in the infection process. The number of assays for monitoring the dynamic process of infection is limited. Here, we describe a novel, label-free, cell-based approach for dynamically monitoring the interaction between bacteria and their hosts.

In recent years, a variety of techniques have emerged as powerful tools for analysis of host-bacterial interaction. Confocal, light, immunofluorescence, and electron microscopy have been used to examine various cytoskeletal components of host cells [4], [5]. Apoptotic, infected cells labeled with cationic dye or annexin-V can be assessed by flow cytometry [23], [24]. Another assay for microbial infection is immunological quantification of the release of mediators into the supernatant [25]. These assays typically analyze a single time point after infection to assess the cellular response, which may lead to incomplete conclusions concerning the process under study. Furthermore, the requirements for extensive labeling, washing, lysing, and fixation limit their applications to small sample sizes. Increasingly, dynamic cell-based assays are important for cellular microbiology since they allow for more dynamic investigation of host-bacterial interaction than currently used methods. Impedance-based technologies use the inherent morphological and adhesive properties of populating cells to yield physiologically relevant and continuous readouts. Time-dependent monitoring of cellular responses upon stimulation with chemical drugs and biological compounds are shown to generate biologically specific profiles [11], [12]. In this work, we further characterized impedance-based, TCRP technology for host-bacterial interaction research.

TCRP is mainly dependent on the following three cellular properties: cell number, morphology and adhesive strength, which are comprehensively associated with cell physiology and are therefore amenable to modulation by real-time, unbiased stimulation [9]. Four studies have described novel applications in microbiology, implying increased attention into cellular responses to bacterial infections [26], [27], [28], [29]. These reports suggest the potential clinical use of impedance-based assay for monitoring cell responses to bacterial infections. Nevertheless, the cellular mechanisms underlying these assays remain largely unidentified. To specifically determine cell-based bacterial infection profiles, we tested intestinal epithelial cell responses to *Salmonella* infection, which is considered to be an important model system for studying the fundamental mechanisms of bacterial infection [4], [30], [31], [32]. We demonstrated that *Salmonella*-induced TCRPs were independent of the attachment of bacteria to electrodes and cell proliferation and showed that only living bacteria and not the LPS, flagellin or conditioned medium induced the TCRPs (Figure 1). Biochemical assays further suggested that these *Salmonella*-induced TCRPs correlated with dynamic cytoskeleton-associated morphological changes, and can be blocked by inhibitors (Figure 3).

**Figure 5:**
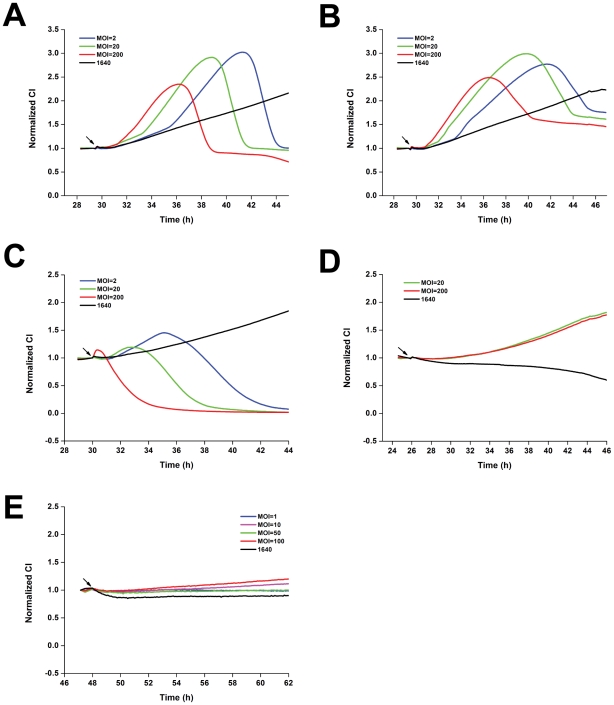
Dynamic monitoring of intestinal epithelial cell responses to intestinal bacteria. HT-29 cells (10,000 cells per well) were seeded into E-plates. The next day, cells were infected with *Y. enterocolitica* (A), *S. flexneri* (B), *L. monocytogenes* (C), *E. coli* DH5α (E), and *Lactobacillus* (F). Arrows, bacterial addition. Representative curves are an average of four replicate wells.

Many pathogenic bacteria invade non-phagocytic cells and result in substantial cytoskeletal rearrangement [33], [34]. As shown in Figure 5A–C, *Yersinia*, *Shegella* and *Listeria* induced an immediate increase in CI following a decline in CI values. However, no two-peak TCRPs were seen in HT-29 cells infected with these intracellular bacteria. We hypothesize that epithelial cells did not regain their normal architecture after bacterial internalization during infection. To further investigate epithelial cell responses to other intestinal bacteria, we also added *Escherichia coli* DH5α and *Lactobacillus to* HT-29 cells. DH5α induced a slow increase in CI that might have resulted from attachment of bacteria to electrodes (Figure 5D). Figure 5F shows that *Lactobacillus* had no effect on HT-29 cells. Based on these different TCRPs, we propose that the TCRP-based assay reflects different cellular responses to different bacteria.

**Figure 6:**
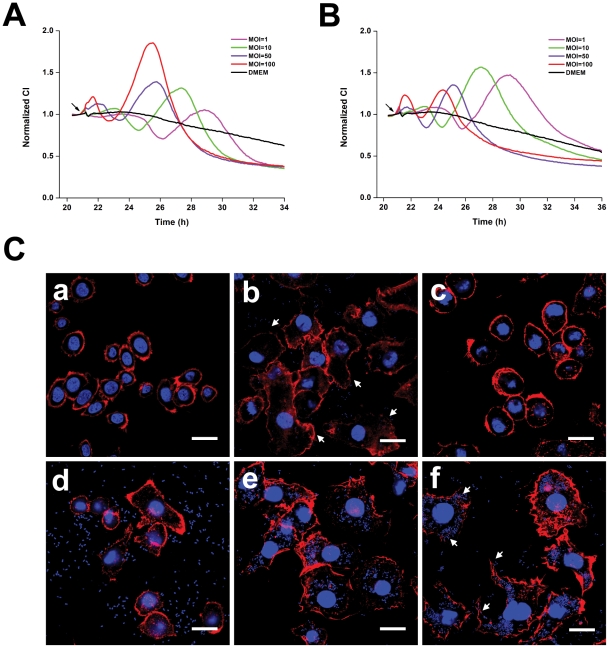
TCRPs and morphological dynamics of macrophages in response to *Salmonella* infection. J774A.1 cells (30,000 cells per well) were seeded into E-plates. The next day, cells were infected with MOIs 1, 10, 50, or 100 of *S. typhimurium* (A) or *S. entertidis* (B). Arrows, bacterial addition. (C) Immunofluorescence of J774A.1 macrophages infected with *S. typhimurium*. (a) Uninfected cells; (b) cells at 45 min post-infection. Arrows, cytoskeleton and membrane ruffling. (c, d) Cells at 1.5 h and 3 h post-infection. Most cells regained its normal architecture. (e, f) Cells at 5 h and 7 h post-infection. Membrane ruffling and *Salmonella*-containing vacuoles (SCV) are shown. Arrows, membrane rupture and release of intracellular bacteria. Representative curves are an average of four replicate wells.

Although an intestinal epithelial cell (HT-29) was used here, TCRP-based assays monitor cellular responses to bacteria and are not limited to a single cell line. Any epithelial cells, immune cells, or even genetically modified cells could be used to meet any specific purposes. The *Salmonella*-induced TCRPs of two other epithelial cell lines were also studied (Figure S4). Similar to HT-29 cells, the TCRPs of both cell lines were dose-dependent. We showed that different cell lines exhibited a variety of TCRPs, reflecting the different natures of bacterial infection. Bacteria can infect a number of immune cell types such as macrophages and dendritic cells, which are critical to both bacteria and host [1], [35]. Therefore, we further investigated the TCRPs produced by macrophages. Figure 6.A and Figure 6.B show the TCRPs produced by J774.A macrophages in response to various MOIs of *S. typhimurium* and *S. enteritidis*. Two serotypes of *Salmonella* induced two-peak TCRPs that were similar as the TCRPs produced by HT-29 cells. Interestingly, the CI deceased to a level below the CI of uninfected cells after the first peak. *Salmonella* invades host macrophages and induces either rapid cell death or establishes an intracellular niche in a *Salmonella*-containing vacuole [35], [36], [37]. We suggest that the transitory CI decease reflects the detachment of some of the macrophages that were undergoing rapid cell death. Therefore, we propose that the TCRP-based technique might also show the details of the competition between bacteria and the immune system components.

*Salmonella* infection of epithelial cells is an excellent model for establishing the TCRP-based method. Moreover, a comparison of the TCRPs induced by different bacteria would be helpful for distinguishing common host responses from species- or pathogen subtype-specific responses. More than 2,500 serovars of *Salmonella* have been reported, which have highly specific host and virulence characteristics [38]. In this paper, we compared two serovars of *Salmonella* and found similar TCRPs (Figure 1). Exposure of cells to bacteria with deficiencies can identify components that are necessary for inducing cellular responses. Using the SV1610 (Dam^−^) strain, we found that the TCRP-based assay might be specifically employed to reveal the function of a bacterial protein critical for pathogenicity.

Traditional antimicrobial compound screening has been established by scoring for growth inhibition *in vitro* on artificial media. Although many classes of antibiotics have been discovered by this method, it does not address the intracellular nature of many pathogens. Many intracellular pathogens are often protected in an intracellular niche from antimicrobial drugs that have either poor permeability or reduced activity in the acidic pH of lysosomes [34], [39]. In this study, an *in vitro* assay was also developed to assess intracellular activity of antibiotic against *Salmonella* using the mouse macrophage cell line J774A.1 (Supplemental Method S1). Gentamycin, enters phagocytes poorly, but ampicillin, chloramphenicol, levofloxacin, and penicillin exhibited intracellular activity (Figure 4E, Table S4). Moreover, *S. typhimurium* SL1344, which is resistant to streptomycin, showed a TCRP identical to control group without antibiotic treatment. Although optimization is needed for this *in vitro* screening model, we suggest that the cell-based assay might help high-throughput development of antimicrobial drugs against intracellular bacteria such as *Shigella*, *Listeria* or *Staphylococcus aureus*.

In the past decade, a variety of technologies involving automation of in vitro bioassays have emerged as powerful tools for high-throughput analysis [40]. The TCRP-based method has been used for drug discovery, providing predictive mechanistic information for small molecule compounds [14]. Although only five antibiotics are investigated in this paper, we show that TCRPs can be useful for studying antibiotic effects in a high-throughput manner. Like other high-throughput methods, the TCRP-based technique requires reliable quantitative measurements and appropriate algorithms to formulate reliable conclusions.

Cell-based TCRP technology that evaluates multiple physiological changes would provide richer information about interactions between bacteria and hosts than endpoint assays [14], [41], [42]. However, the inherent variability and complexity of cells should be taken into full consideration in experimental design, implementation, and analysis. Introduction of statistical model of TCRPs will address this problem [41]. In addition, because of the ability to measure over time and dosage, TCRP offers the possibility of using multiple variables in mathematic simulations for kinetic measurement of cell phenotypic changes. We believe that in the near future, TCRP integrated with statistical analyses would aid in the analysis of host-pathogenic interactions.

In this paper, we report a label-free, cell-based method for dynamically monitoring the host-bacterial interactions. Research focusing on the host-bacterial interaction has been hampered because of the limited tools for effective analysis. The TCRP-based methodology, together with other emerging advances in cell-based assays, might overcome these problems to give clearer insights into the interaction between bacteria and their hosts.

## Materials and Methods

### Cells and bacteria

The human epithelial cell lines HT-29, SW480, HCT116 were from the Cell Bank of Type Culture of Chinese Academy of Sciences (CBTCCAS, Shanghai, China) and maintained in RPMI 1640 medium (Hyclone) containing 10% fetal bovine serum (GIBCO) at 37°C and 5% CO_2_. *S. enterica* serovar Typhimurium wild type strain SL1344 and its mutant strain SV1610 (Dam^−^) were kindly provided by Dr. W.H Fang (School of Animal Science, Zhejiang University). *S. enterica* serovar Enteritidis, *Yersinia enterocolitica*, *Shigella flexneri*, *Listeria monocytogenes*, *Enterohaemorrhagic E. coli (EHEC)*, and *Lactobacillus* were from National Center for Medical Culture Collection (CMCC, Beijing, China). *E. coli* DH5α was from Tiangen Biotech (Beijing, China). All bacteria were cultured overnight in 5 ml of recommended bacterial culture medium without agitation at 37°C, diluted 1∶50 in medium and cultured approximately 6 h without agitation at 37°C. Before inoculation, bacteria were harvested by centrifugation and washed with phosphate buffer saline (PBS) and finally suspended in cell culture medium. The optical density (OD) of bacterial suspensions was determined at 600 nm, and the bacteria per ml was calculated based on 1 OD600 nm = 1×10^9^ CFU/ml.

### xCELLigence system

The xCELLigence system (Roche Applied Science) has three components: an analyzer, a device station and a 16-well or 96-well E-plate. The electronic impedance of each well was continuously measured and recorded. The dimensionless parameter, CI was derived based on measured impedance values, as:
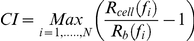
where 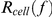 and 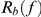 are the frequency-dependent electrode impedance with or without cells present, respectively and *N* is the number of the frequency points at which the impedance was measured. Thus, CI is a quantitative measure of cell status in a well, reflecting the biological status of monitored cells, including the cell number, cell viability, adhesion degree and morphology [13], [43], [44]. Normalization by dividing CI readouts of all wells at all time points by the CI of individual wells at a normalization time point allowed cell index comparisons between the wells [13].

### RTCA experimental setup and bacterial infection assay

Detailed RTCA experimental procedures were as described previously [9], [45], [46]. Briefly, 100 µl of medium were added to wells in the E-plate to obtain background readings followed by the addition of 100 µl of cell suspension containing the indicated number of cells. For the bacterial infection assay, cells were seeded into an E-plate and incubated at 37°C. Before inoculation, cells were washed twice with PBS, and incubated in serum-free medium for 3 h. After starvation, the electronic sensor analyzer was discontinued and various MOIs of bacteria, suspended in 10 µl media, were added to the wells. Analysis was resumed and CIs were measured every 5 min. The data are presented as CI normalized to the last time point before bacterial addition. All data in this work were from a minimum of four multiple-well duplicates and characterized by TCRP-based analysis.

### Cytotoxicity assays

HT-29 cells were seeded onto a 96-well plate at 10,000 cells per well and incubated for 24 h at 37°C. Before infection with the bacteria, the medium was replaced with serum-free RPMI 1640 medium. Infection was as described above. At the indicated time points during the infections, culture supernatants were collected and centrifuged at 13,000× *g* to remove bacteria. Cytotoxicity was quantified colorimetrically with the CytoTox96 Non-Radioactive Cytotoxicity Assay (Promega, Madison, WI). The percentage of cytotoxicity was calculated using the formula: 100%×([experimental release – spontaneous release]/[maximum release – spontaneous release]), in which spontaneous release was the amount of LDH activity in the supernatant of uninfected cells and total release was the activity in HT-29 cell lysates.

### Apoptosis assay

Adherent cells were suspended and collected with non-adherent cells by centrifugation. FITC-conjugated annexin-V and propidium iodide (PI) were added to approximately 1×10^5^ cells according to the manufacturer's instruction (Apoptosis Detection Kit, Beyotime, Jiangsu, China). Early apoptotic cells were stained with annexin-V alone, whereas necrotic cells and late apoptotic cells were stained with both annexin-V and PI. Stained cells were analyzed with a flow cytometer (Beckman).

### Cell morphology analysis by fluorescence microscopy

Cell morphological changes following *S. typhimurium* infection were examined by fluorescence microscopy. HT-29 cells were seeded into 24-well tissue culture plates containing glass coverslips. An infection assay was performed as described above. Coverslips were washed with PBS and fixed in 4% paraformaldehyde for 10 min. Fixed cells were washed three times with PBS, permeablized in 0.1% Triton-100 (v/v) in PBS, and blocked using 1% bovine serum albumin (BSA) in PBS. Cells were stained with TRITC-phalloidin for 30 min, washed three times with PBS, and stained with DAPI for 5 min. Stained cells were visualized and photographed with a Zeiss LSM 510 confocal fluorescence microscope. Images were merged and analyzed using LSM Image Browser software (Zeiss).

### Gentamycin protection assay

The number of invasive bacteria was determined as described previously [4]. HT-29 cells were seeded into a 24-well plate at a density of 50,000 cells per well and infected with bacteria as described above. After 1 h of infection, medium was replaced with fresh medium containing 50 µg/ml gentamycin. After 1 h of incubation, cells were washed three times with PBS and lysed with 0.1% Triton X-100 for 15 min. Dilutions were plated on LB agar to determine the number of recovered viable bacteria.

### Immunobloting analysis

HT-29 cells were seeded into a 6-well plate and infected with 200 MOI of *S. typhimurium* SL 1344. At 0, 0.75, 1.5, 3, 5, 7 and 9 h post-infection, cells were lysed with radioimmunoprecipitation assay (RIPA) buffer containing protease inhibitors, and the lysate was centrifuged for 10 min at 13,000× *g*. Western blotting was performed and membranes were immunoprobed with antibodies against Rac1/2/3, RhoA, and WASP as previously described [18].

## Supporting Information

Figure S1 **Dynamic monitoring of intestinal epithelial cell response to** ***S. entertidis*** **infection.** (A) HT-29 cells (10,000 cells per well) were seeded into E-plates. After approximately 46 h, the cells were infected with MOIs 0.1, 1, 10, 50, 100, or 200 of *S. entertidis*. (B) Inoculums of 5,000, 10,000, or 20,000 cells per well of HT-29 cells were seeded into E-plate wells. Fixed amounts (2×10^6^ cfu) of *S. entertidis* were added and monitored. arrows, bacterial addition. Representative curves are an average of four replicate wells.
(TIF)

Figure S2 **Sequential morphological changes of cells infected with** ***Salmonella*** **visualized by scanning electron microscopy (SEM).** HT-29 cells were seeded into 24-well plates with cover slides and infected with SL1344 (MOI = 200). Cells were fixed at 0 (a), 0.75 (b), 1.5 (c), 3 (d), 5 (e), and 7 (f) h post infection and observed by SEM. Scale bar, 20 µm. Arrows, pseudopodia extension and membrane ruffling induced by *Salmonella*.
(TIF)

Figure S3 **Microarray analysis of HT-29 cellular responses to** ***Salmonella*** **infection.** (A) Scatter plot of infected samples (45 min, 3 h, and 7 h post-infection) vs. uninfected sample (0 h). Each dot represents a normalized intensity of a probe sets. (B) CV curve for identifying significantly differently expressed genes. Each probe set is presented as a circle. Probe sets with significantly different expression with CV values greater than the calculated cut-off (0.3572) are in red, and probe sets with CV values less than the cut-off are in blue. (C) Transcriptional response of HT-29 epithelial cells to *Salmonella* infection. Cells were treated with *S. typhimurium* (ATCC SL1344) for 0 min, 45 min, 3 h, or 7 h. Data represent a time course of expression profiles of 272 probe sets with significantly different expression (Table S1). Expression patterns of individual probe sets were organized by unsupervised clustering. Data are a matrix with each row representing an individual probe set, and each column an experimental condition. Blue, relatively low-level expression; yellow, high-level expression (see scale). Color intensity corresponds to the logarithmically normalized expression intensity.
(TIF)

Figure S4 **Different cell lines show different TCRPs of** ***Salmonella*** **infection.** (A) TCRPs of HCT116 cells in response to *S. typhimurium* infection. (B) TCRPs of SW480 cells in response to *S. typhimurium* infection. Arrows, bacterial addition. Representative curves are an average of four replicate wells.
(TIF)

Table S1 **Genes with significantly different expression during infection (272 total).**
(DOC)

Table S2 **Gene ontology terms most highly represented in genes with significantly different expression.**
(DOC)

Table S3 **Pathways in response of HT29 epithelia to** ***S. typhimurium*** **.**
(DOC)

Table S4 **MICs of eight antibiotics for** ***Salmonella*** **strain SL1344.**
(DOC)

Supplemental Methods S1 (DOC)
