# Supplementary figures and images for: Phenotypic Pattern-Based Assay for Dynamically Monitoring Host Cellular Responses to Salmonella Infections
Source: PLoS One. 2011 Nov 3;6(11):e26544. doi: 10.1371/journal.pone.0026544 (PMC3207827; doi:10.1371/journal.pone.0026544)

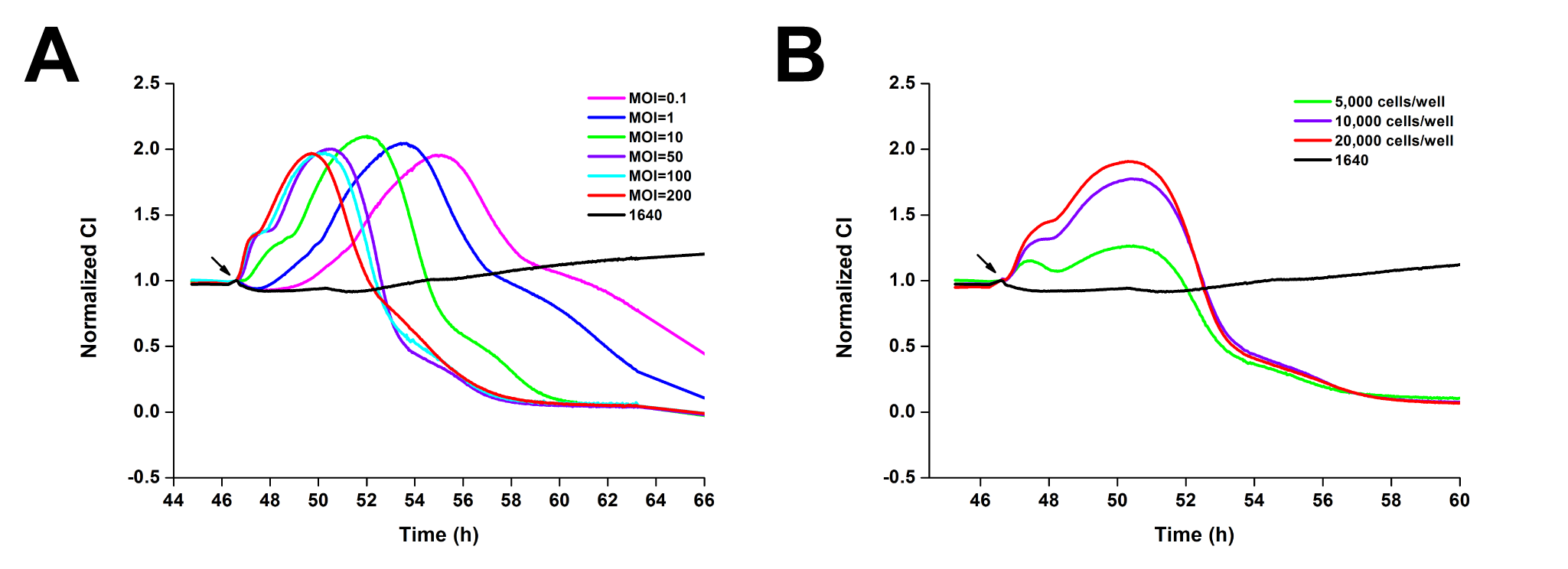

Supplement: Figure S1 — Dynamic monitoring of intestinal epithelial cell response to S. entertidis infection. (A) HT-29 cells (10,000 cells per well) were seeded into E-plates. After approximately 46 h, the cells were infected with MOIs 0.1, 1, 10, 50, 100, or 200 of S. entertidis. (B) Inoculums of 5,000, 10,000, or 20,000 cells per well of HT-29 cells were seeded into E-plate wells. Fixed amounts (2×10^6^ cfu) of S. entertidis were added and monitored. arrows, bacterial addition. Representative curves are an average of four replicate wells. (TIF) [file pone.0026544.s001.tif]

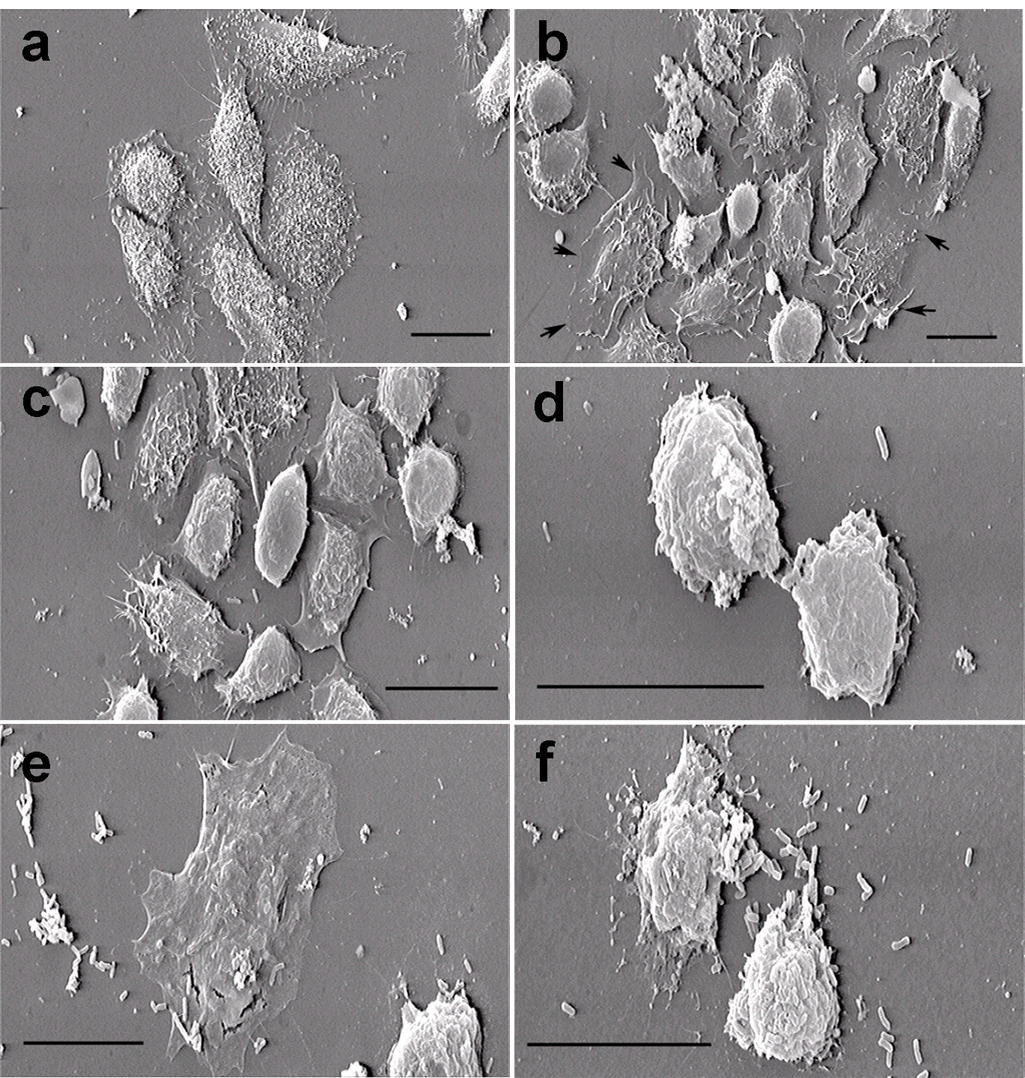

Supplement: Figure S2 — Sequential morphological changes of cells infected with Salmonella visualized by scanning electron microscopy (SEM). HT-29 cells were seeded into 24-well plates with cover slides and infected with SL1344 (MOI = 200). Cells were fixed at 0 (a), 0.75 (b), 1.5 (c), 3 (d), 5 (e), and 7 (f) h post infection and observed by SEM. Scale bar, 20 µm. Arrows, pseudopodia extension and membrane ruffling induced by Salmonella. (TIF) [file pone.0026544.s002.tif]

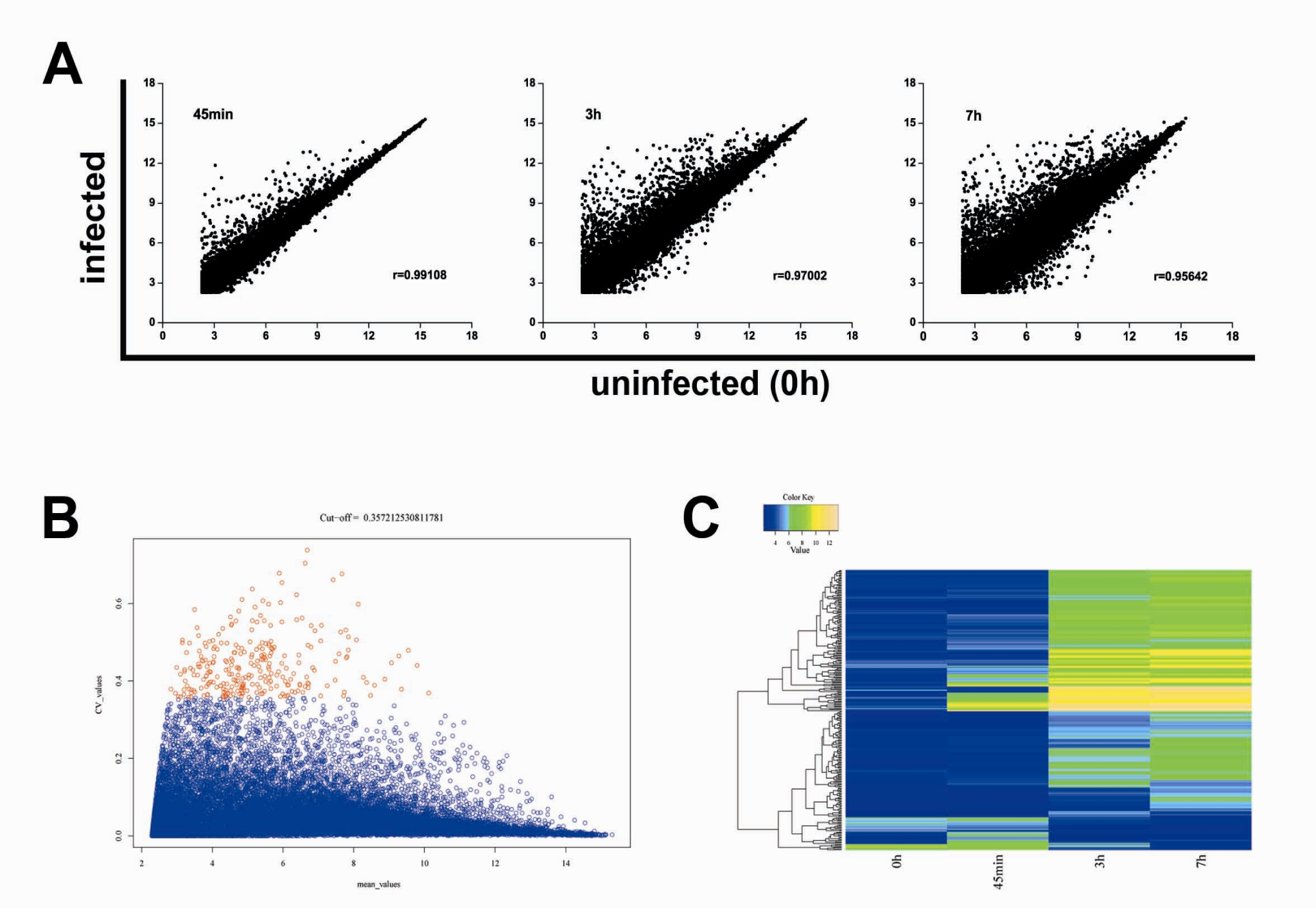

Supplement: Figure S3 — Microarray analysis of HT-29 cellular responses to Salmonella infection. (A) Scatter plot of infected samples (45 min, 3 h, and 7 h post-infection) vs. uninfected sample (0 h). Each dot represents a normalized intensity of a probe sets. (B) CV curve for identifying significantly differently expressed genes. Each probe set is presented as a circle. Probe sets with significantly different expression with CV values greater than the calculated cut-off (0.3572) are in red, and probe sets with CV values less than the cut-off are in blue. (C) Transcriptional response of HT-29 epithelial cells to Salmonella infection. Cells were treated with S. typhimurium (ATCC SL1344) for 0 min, 45 min, 3 h, or 7 h. Data represent a time course of expression profiles of 272 probe sets with significantly different expression (Table S1). Expression patterns of individual probe sets were organized by unsupervised clustering. Data are a matrix with each row representing an individual probe set, and each column an experimental condition. Blue, relatively low-level expression; yellow, high-level expression (see scale). Color intensity corresponds to the logarithmically normalized expression intensity. (TIF) [file pone.0026544.s003.tif]

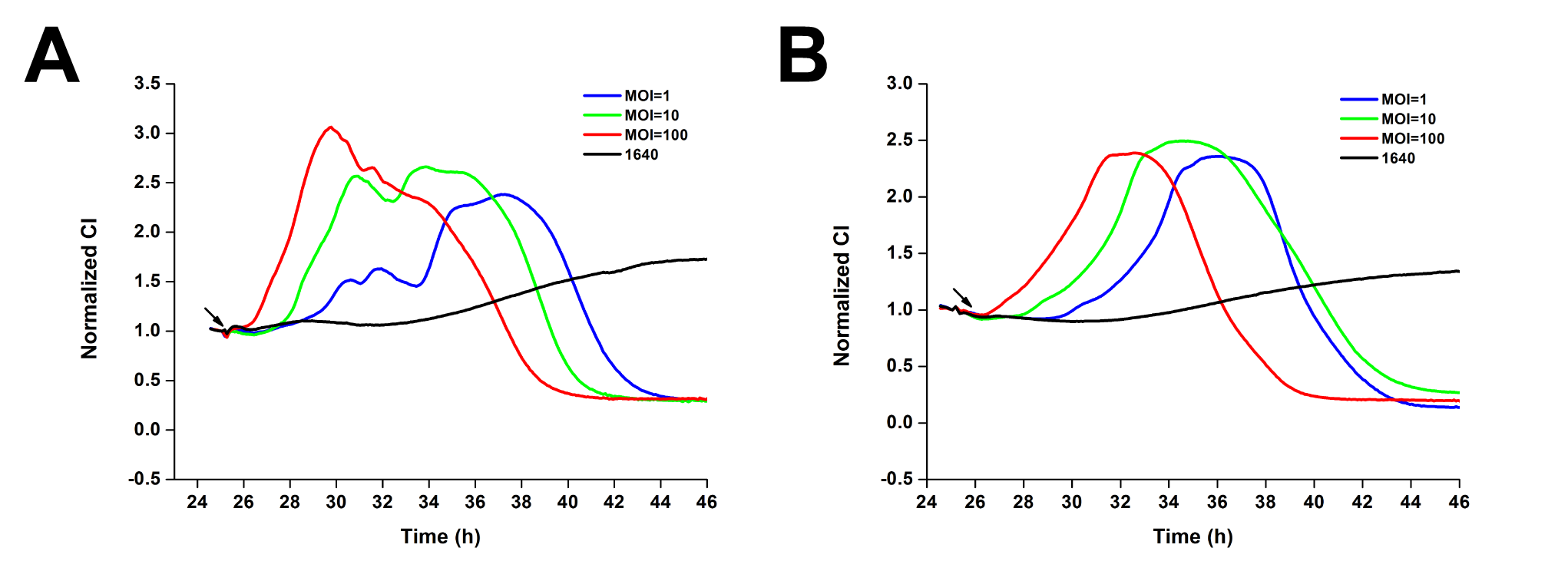

Supplement: Figure S4 — Different cell lines show different TCRPs of Salmonella infection. (A) TCRPs of HCT116 cells in response to S. typhimurium infection. (B) TCRPs of SW480 cells in response to S. typhimurium infection. Arrows, bacterial addition. Representative curves are an average of four replicate wells. (TIF) [file pone.0026544.s004.tif]
