# Supplementary material for: Phenotypic Pattern-Based Assay for Dynamically Monitoring Host Cellular Responses to Salmonella Infections
Source: PLoS One. 2011 Nov 3;6(11):e26544. doi: 10.1371/journal.pone.0026544 (PMC3207827; doi:10.1371/journal.pone.0026544)
Supplement: Table S1 — Genes with significantly different expression during infection (272 total). (DOC) [file pone.0026544.s005.doc]

**Table S1. Genes with significantly different Expression during i**nfection (272 total)

| **Probe** | **Symbol** | **Description** | **Normalized Intensity** | | | |
| --- | --- | --- | --- | --- | --- | --- |
|  |  |  | **0h** | **45min** | **3h** | **7h** |
| **1552258_at** | NCRNA00152 | non-protein coding RNA 152 | 2.505567 | 4.070258 | 7.304224 | 7.211926 |
| **1552326_a_at** | CCDC11 | coiled-coil domain containing 11 | 2.281685 | 2.301409 | 3.023908 | 5.80149 |
| **1552362_a_at** | LEAP2 | liver expressed antimicrobial peptide 2 | 2.591978 | 3.074016 | 5.703355 | 6.884827 |
| **1552685_a_at** | GRHL1 | grainyhead-like 1 (Drosophila) | 3.243222 | 3.471411 | 6.580484 | 7.947761 |
| **1552999_a_at** | WFDC10B | WAP four-disulfide core domain 10B | 2.782796 | 2.281725 | 3.60058 | 6.505695 |
| **1554283_at** | CCRN4L | CCR4 carbon catabolite repression 4-like (S. cerevisiae) | 2.85604 | 3.659033 | 6.033973 | 7.20003 |
| **1554588_a_at** | TTC30B | tetratricopeptide repeat domain 30B | 4.843125 | 3.200192 | 2.376675 | 2.357642 |
| **1554703_at** | ARHGEF10 | Rho guanine nucleotide exchange factor (GEF) 10 | 2.565633 | 2.617744 | 4.713859 | 5.15001 |
| **1554980_a_at** | ATF3 | activating transcription factor 3 | 3.031821 | 5.99992 | 11.14642 | 11.21517 |
| **1555370_a_at** | CAMTA1 | calmodulin binding transcription activator 1 | 2.336184 | 3.156611 | 6.406436 | 6.565508 |
| **1555673_at** |  |  | 5.453192 | 3.773286 | 10.89033 | 12.18203 |
| **1555858_at** |  |  | 2.580407 | 5.013379 | 2.603582 | 2.603582 |
| **1555862_s_at** | MICALL2 | MICAL-like 2 | 3.080259 | 2.610539 | 7.87751 | 8.301149 |
| **1556049_at** | RTN4 | reticulon 4 | 2.624755 | 3.902734 | 7.856498 | 8.042667 |
| **1556066_at** | KDM6B | lysine (K)-specific demethylase 6B | 3.18384 | 4.892233 | 9.187163 | 8.523784 |
| **1556067_a_at** | KDM6B | lysine (K)-specific demethylase 6B | 3.305116 | 3.305116 | 9.778396 | 9.147969 |
| **1556113_at** | DKFZp451A211 | DKFZp451A211 protein | 2.463995 | 2.463995 | 5.39715 | 5.175434 |
| **1556114_a_at** | DKFZp451A211 | DKFZp451A211 protein | 3.247178 | 3.268846 | 7.517985 | 7.5044 |
| **1556220_at** |  |  | 2.281685 | 2.588025 | 6.095647 | 7.175914 |
| **1556221_a_at** |  |  | 3.728182 | 4.78261 | 7.542295 | 8.364884 |
| **1557050_at** |  |  | 3.2527 | 4.303388 | 6.301682 | 7.64674 |
| **1557512_at** |  |  | 2.53923 | 2.432489 | 5.018716 | 3.341109 |
| **1557521_a_at** |  |  | 2.501608 | 4.414164 | 7.527803 | 8.192743 |
| **1557522_x_at** |  |  | 3.283334 | 5.491203 | 7.591663 | 8.193788 |
| **1557555_at** |  |  | 3.535703 | 3.535703 | 7.484406 | 4.955401 |
| **1558365_at** |  |  | 3.109781 | 3.005853 | 5.25596 | 6.19337 |
| **1558378_a_at** | AHNAK2 | AHNAK nucleoprotein 2 | 2.281685 | 2.281685 | 5.360241 | 6.134363 |
| **1558404_at** | LOC644242 | hypothetical protein LOC644242 | 2.495515 | 2.369709 | 9.542382 | 9.159639 |
| **1561914_at** |  |  | 2.782425 | 6.545662 | 2.378365 | 2.281685 |
| **1562144_at** |  |  | 3.00719 | 3.048689 | 5.967746 | 5.718286 |
| **1562747_at** |  |  | 2.46011 | 2.46011 | 5.260764 | 6.384584 |
| **1568732_at** |  |  | 2.281685 | 2.281685 | 4.006552 | 4.797945 |
| **1568765_at** | SERPINE1 | serpin peptidase inhibitor, clade E (nexin, plasminogen activator inhibitor type 1), member 1 | 2.952301 | 7.218642 | 10.18256 | 10.80494 |
| **1569065_s_at** | C15orf62 | chromosome 15 open reading frame 62 | 2.851761 | 3.114503 | 6.394284 | 6.648606 |
| **1569263_at** |  |  | 3.193438 | 3.321928 | 6.887559 | 6.067434 |
| **1569477_at** |  |  | 2.37933 | 4.469663 | 6.669374 | 4.700589 |
| **1570061_at** |  |  | 2.843243 | 2.663502 | 5.557293 | 5.426972 |
| **201169_s_at** | BHLHE40 | basic helix-loop-helix family, member e40 | 2.83299 | 2.655209 | 7.729546 | 7.827497 |
| **201465_s_at** | JUN | jun oncogene | 4.355481 | 9.312783 | 11.71428 | 12.0131 |
| **201693_s_at** | EGR1 | early growth response 1 | 2.965821 | 10.07575 | 11.76459 | 12.29727 |
| **201860_s_at** | PLAT | plasminogen activator, tissue | 3.258259 | 2.282493 | 3.642092 | 6.231697 |
| **202340_x_at** | NR4A1 | nuclear receptor subfamily 4, group A, member 1 | 3.970635 | 9.091625 | 10.68832 | 10.14994 |
| **202464_s_at** | PFKFB3 | 6-phosphofructo-2-kinase/fructose-2,6-biphosphatase 3 | 3.747567 | 4.324901 | 8.197916 | 8.922375 |
| **202478_at** | TRIB2 | tribbles homolog 2 (Drosophila) | 8.010083 | 7.899638 | 5.647735 | 2.944116 |
| **202510_s_at** | TNFAIP2 | tumor necrosis factor, alpha-induced protein 2 | 3.700089 | 9.198793 | 11.19318 | 11.42159 |
| **202643_s_at** | TNFAIP3 | tumor necrosis factor, alpha-induced protein 3 | 2.281685 | 6.330434 | 9.369311 | 9.17939 |
| **202644_s_at** | TNFAIP3 | tumor necrosis factor, alpha-induced protein 3 | 2.47548 | 7.988222 | 10.58607 | 10.32352 |
| **202668_at** | EFNB2 | ephrin-B2 | 2.942944 | 3.09562 | 5.401785 | 6.008897 |
| **202859_x_at** | IL8 | interleukin 8 | 2.314726 | 6.857764 | 10.63648 | 11.29607 |
| **203518_at** | LYST | lysosomal trafficking regulator | 2.610902 | 2.593888 | 4.756048 | 7.037177 |
| **203821_at** | HBEGF | heparin-binding EGF-like growth factor | 3.115786 | 4.134862 | 9.661252 | 10.26758 |
| **203879_at** | PIK3CD | phosphoinositide-3-kinase, catalytic, delta polypeptide | 4.129159 | 3.517035 | 8.11046 | 8.609889 |
| **204470_at** | CXCL1 | chemokine (C-X-C motif) ligand 1 (melanoma growth stimulating activity, alpha) | 2.370801 | 8.211012 | 9.650218 | 10.09391 |
| **204472_at** | GEM | GTP binding protein overexpressed in skeletal muscle | 2.738772 | 3.642048 | 11.23718 | 12.05356 |
| **204657_s_at** | SHB | Src homology 2 domain containing adaptor protein B | 2.281685 | 2.390947 | 3.899356 | 5.344506 |
| **204729_s_at** | STX1A | syntaxin 1A (brain) | 3.352986 | 3.985962 | 5.803803 | 7.943639 |
| **204743_at** | TAGLN3 | transgelin 3 | 2.376431 | 2.812968 | 4.564952 | 6.301825 |
| **204760_s_at** |  |  | 3.227801 | 3.372391 | 6.970566 | 8.287106 |
| **204908_s_at** | BCL3 | B-cell CLL/lymphoma 3 | 2.834245 | 4.868271 | 6.844788 | 7.121686 |
| **204912_at** | IL10RA | interleukin 10 receptor, alpha | 2.281685 | 2.366577 | 3.485453 | 5.007596 |
| **205239_at** | AREG | amphiregulin | 2.601315 | 2.601315 | 7.682808 | 8.828825 |
| **205476_at** | CCL20 | chemokine (C-C motif) ligand 20 | 2.348411 | 5.622191 | 9.594912 | 10.78077 |
| **205479_s_at** | PLAU | plasminogen activator, urokinase | 4.45956 | 7.303031 | 11.06439 | 11.52734 |
| **205547_s_at** | TAGLN | transgelin | 2.947866 | 3.045079 | 6.172593 | 6.24965 |
| **205579_at** | HRH1 | histamine receptor H1 | 2.281685 | 2.281685 | 7.236328 | 7.552592 |
| **205580_s_at** | HRH1 | histamine receptor H1 | 2.819645 | 2.662229 | 6.193899 | 6.469936 |
| **205599_at** | TRAF1 | TNF receptor-associated factor 1 | 2.735353 | 3.058665 | 4.954333 | 6.790595 |
| **205659_at** | HDAC9 | histone deacetylase 9 | 2.758492 | 2.94981 | 5.551822 | 5.998374 |
| **205822_s_at** | HMGCS1 | 3-hydroxy-3-methylglutaryl-Coenzyme A synthase 1 (soluble) | 2.353409 | 2.843389 | 4.284662 | 6.776125 |
| **205871_at** |  |  | 2.442864 | 3.366288 | 5.825357 | 6.299313 |
| **205931_s_at** | CREB5 | cAMP responsive element binding protein 5 | 2.306545 | 2.306545 | 3.557933 | 6.267845 |
| **205945_at** | IL6R | interleukin 6 receptor | 2.719335 | 2.668514 | 8.560748 | 8.416327 |
| **206337_at** | CCR7 | chemokine (C-C motif) receptor 7 | 2.870042 | 2.315583 | 10.35224 | 10.953 |
| **206374_at** | DUSP8 | dual specificity phosphatase 8 | 3.994024 | 4.177421 | 7.912098 | 7.862842 |
| **206411_s_at** | ABL2 | v-abl Abelson murine leukemia viral oncogene homolog 2 (arg, Abelson-related gene) | 2.281685 | 2.281685 | 4.047319 | 5.910228 |
| **206432_at** | HAS2 | hyaluronan synthase 2 | 4.964289 | 4.769921 | 2.30854 | 2.281685 |
| **206569_at** | IL24 | interleukin 24 | 2.425167 | 2.41185 | 10.60332 | 11.29626 |
| **206758_at** | EDN2 | endothelin 2 | 3.454438 | 3.7909 | 8.090735 | 8.819473 |
| **206801_at** | NPPB | natriuretic peptide precursor B | 2.366226 | 2.366226 | 2.366226 | 5.514716 |
| **206825_at** | OXTR | oxytocin receptor | 2.557899 | 2.557899 | 3.877652 | 6.224923 |
| **206848_at** |  |  | 5.827084 | 4.369405 | 2.562836 | 2.553052 |
| **206924_at** | IL11 | interleukin 11 | 3.386943 | 2.982951 | 11.72289 | 12.56891 |
| **206926_s_at** | IL11 | interleukin 11 | 2.536111 | 2.985524 | 6.377196 | 7.039502 |
| **207147_at** | DLX2 | distal-less homeobox 2 | 2.940393 | 4.930078 | 7.263336 | 7.496472 |
| **207442_at** | CSF3 | colony stimulating factor 3 (granulocyte) | 3.377494 | 3.377494 | 4.095147 | 7.355159 |
| **207536_s_at** | TNFRSF9 | tumor necrosis factor receptor superfamily, member 9 | 2.8323 | 2.8323 | 5.813946 | 6.679805 |
| **207708_at** | ALOXE3 | arachidonate lipoxygenase 3 | 2.786248 | 2.910019 | 6.583986 | 6.927984 |
| **207746_at** | POLQ | polymerase (DNA directed), theta | 4.72155 | 5.600302 | 2.337207 | 2.281685 |
| **207767_s_at** | EGR4 | early growth response 4 | 2.281685 | 6.207908 | 8.888759 | 8.41203 |
| **207768_at** | EGR4 | early growth response 4 | 2.745265 | 7.683608 | 10.63282 | 10.1853 |
| **207850_at** | CXCL3 | chemokine (C-X-C motif) ligand 3 | 3.248674 | 9.268952 | 10.07669 | 10.48776 |
| **207876_s_at** | FLNC | filamin C, gamma | 5.006952 | 4.294641 | 7.260591 | 9.676443 |
| **208300_at** | PTPRH | protein tyrosine phosphatase, receptor type, H | 3.134603 | 3.395245 | 5.492604 | 6.834397 |
| **209294_x_at** | TNFRSF10B | tumor necrosis factor receptor superfamily, member 10b | 3.819065 | 2.942114 | 5.833924 | 6.699167 |
| **209324_s_at** | RGS16 | regulator of G-protein signaling 16 | 2.741802 | 3.539341 | 6.505481 | 5.968353 |
| **209457_at** | DUSP5 | dual specificity phosphatase 5 | 5.489633 | 8.701274 | 12.97061 | 13.31736 |
| **209774_x_at** | CXCL2 | chemokine (C-X-C motif) ligand 2 | 3.421597 | 10.89809 | 12.26296 | 12.59079 |
| **209959_at** | NR4A3 | nuclear receptor subfamily 4, group A, member 3 | 2.633191 | 2.330675 | 5.363519 | 5.351385 |
| **210090_at** | ARC | activity-regulated cytoskeleton-associated protein | 4.31574 | 3.016538 | 7.440034 | 7.885031 |
| **210164_at** | GZMB | granzyme B (granzyme 2, cytotoxic T-lymphocyte-associated serine esterase 1) | 3.58417 | 3.696355 | 7.207788 | 7.94679 |
| **210229_s_at** | CSF2 | colony stimulating factor 2 (granulocyte-macrophage) | 3.780555 | 7.961051 | 13.14774 | 13.28997 |
| **210405_x_at** | TNFRSF10B | tumor necrosis factor receptor superfamily, member 10b | 3.093937 | 3.014355 | 5.976148 | 6.818733 |
| **211506_s_at** | IL8 | interleukin 8 | 2.46482 | 6.446171 | 10.48016 | 11.12208 |
| **211527_x_at** | VEGFA | vascular endothelial growth factor A | 3.554661 | 3.57974 | 7.115871 | 7.090791 |
| **211668_s_at** | PLAU | plasminogen activator, urokinase | 2.922975 | 3.841829 | 9.317927 | 10.08328 |
| **212019_at** | RSL1D1 | ribosomal L1 domain containing 1 | 4.015673 | 2.658323 | 6.284693 | 6.133846 |
| **212135_s_at** | ATP2B4 | ATPase, Ca++ transporting, plasma membrane 4 | 2.888484 | 2.281685 | 3.403816 | 6.144143 |
| **212641_at** | HIVEP2 | human immunodeficiency virus type I enhancer binding protein 2 | 3.141398 | 2.957922 | 7.100273 | 7.293201 |
| **212764_at** | ZEB1 | zinc finger E-box binding homeobox 1 | 3.463696 | 3.567754 | 6.615179 | 7.815833 |
| **213139_at** | SNAI2 | snail homolog 2 (Drosophila) | 4.068301 | 3.778078 | 11.99827 | 12.66442 |
| **213146_at** | KDM6B | lysine (K)-specific demethylase 6B | 2.366996 | 4.45259 | 8.128113 | 7.749607 |
| **213268_at** | CAMTA1 | calmodulin binding transcription activator 1 | 2.735903 | 2.63296 | 4.512716 | 5.923734 |
| **213352_at** | TMCC1 | transmembrane and coiled-coil domain family 1 | 3.42468 | 3.095584 | 5.758919 | 7.055137 |
| **213557_at** | CRKRS | Cdc2-related kinase, arginine/serine-rich | 2.637701 | 3.273389 | 5.509206 | 5.868049 |
| **214056_at** | MCL1 | myeloid cell leukemia sequence 1 (BCL2-related) | 2.597786 | 3.590696 | 7.204084 | 7.031689 |
| **214123_s_at** | C4orf10 | chromosome 4 open reading frame 10 | 3.967379 | 2.755256 | 7.912313 | 8.179769 |
| **214185_at** | KHDRBS1 | KH domain containing, RNA binding, signal transduction associated 1 | 2.293101 | 2.293101 | 4.612766 | 5.609803 |
| **214234_s_at** |  |  | 2.410976 | 3.255802 | 6.603236 | 7.052201 |
| **214291_at** |  |  | 3.053945 | 3.528051 | 4.266084 | 6.591803 |
| **214782_at** | CTTN | cortactin | 2.763162 | 4.417621 | 6.353545 | 6.602906 |
| **214925_s_at** | SPTAN1 | spectrin, alpha, non-erythrocytic 1 (alpha-fodrin) | 2.434106 | 4.891049 | 5.781587 | 6.808182 |
| **214985_at** | EXT1 | exostoses (multiple) 1 | 2.577918 | 2.585556 | 4.712462 | 5.46251 |
| **215069_at** | NMT2 | N-myristoyltransferase 2 | 4.160439 | 4.228743 | 6.736683 | 8.575364 |
| **215188_at** | STK24 | serine/threonine kinase 24 (STE20 homolog, yeast) | 2.934777 | 2.929883 | 6.286631 | 6.638349 |
| **215231_at** | PRKAG2 | protein kinase, AMP-activated, gamma 2 non-catalytic subunit | 2.281685 | 2.728735 | 4.905373 | 6.271623 |
| **215501_s_at** | DUSP10 | dual specificity phosphatase 10 | 3.585709 | 3.065996 | 5.95132 | 6.716651 |
| **215599_at** |  |  | 5.909256 | 6.364743 | 4.058894 | 2.281685 |
| **215743_at** | NMT2 | N-myristoyltransferase 2 | 3.035355 | 3.435692 | 7.34642 | 8.56959 |
| **217173_s_at** | LDLR | low density lipoprotein receptor | 3.381115 | 5.317488 | 8.230163 | 8.074817 |
| **217482_at** |  |  | 2.491318 | 2.492032 | 4.680444 | 2.492032 |
| **217584_at** | NPC1 | Niemann-Pick disease, type C1 | 2.782424 | 2.518766 | 4.90499 | 5.251339 |
| **218689_at** | FANCF | Fanconi anemia, complementation group F | 7.107186 | 6.576578 | 4.110197 | 2.617011 |
| **218810_at** | ZC3H12A | zinc finger CCCH-type containing 12A | 3.739719 | 7.576046 | 10.07753 | 9.539087 |
| **218964_at** | ARID3B | AT rich interactive domain 3B (BRIGHT-like) | 4.294939 | 4.435124 | 8.825802 | 9.972983 |
| **219181_at** | LIPG | lipase, endothelial | 3.370767 | 3.687662 | 7.325483 | 8.016623 |
| **219423_x_at** | TNFRSF25 | tumor necrosis factor receptor superfamily, member 25 | 2.614316 | 2.281685 | 3.860944 | 5.151077 |
| **219500_at** | CLCF1 | cardiotrophin-like cytokine factor 1 | 3.005331 | 4.605632 | 7.935263 | 7.730744 |
| **219538_at** | WDR5B | WD repeat domain 5B | 5.953263 | 5.250624 | 3.271993 | 2.711453 |
| **219947_at** | CLEC4A | C-type lectin domain family 4, member A | 2.331935 | 2.333065 | 2.958627 | 5.238862 |
| **220370_s_at** | USP36 | ubiquitin specific peptidase 36 | 4.195825 | 4.345035 | 9.17766 | 9.632081 |
| **220468_at** | ARL14 | ADP-ribosylation factor-like 14 | 2.31339 | 2.281685 | 7.8637 | 8.056179 |
| **220709_at** | ZNF556 | zinc finger protein 556 | 3.12096 | 3.12224 | 4.446447 | 6.59061 |
| **221009_s_at** | ANGPTL4 | angiopoietin-like 4 | 2.979197 | 2.979197 | 9.027943 | 10.53706 |
| **221563_at** | DUSP10 | dual specificity phosphatase 10 | 2.299893 | 3.557975 | 6.82688 | 7.580708 |
| **221617_at** | TAF9B | TAF9B RNA polymerase II, TATA box binding protein (TBP)-associated factor, 31kDa | 3.023567 | 3.264687 | 6.861393 | 8.48212 |
| **221667_s_at** | HSPB8 | heat shock 22kDa protein 8 | 3.046259 | 3.47888 | 6.911303 | 8.131585 |
| **221973_at** |  |  | 2.541528 | 3.427876 | 5.676757 | 5.851344 |
| **222164_at** | FGFR1 | fibroblast growth factor receptor 1 | 4.681122 | 6.002981 | 9.996253 | 10.16281 |
| **222383_s_at** | ALOXE3 | arachidonate lipoxygenase 3 | 2.281685 | 2.587218 | 6.615211 | 7.185653 |
| **222891_s_at** | BCL11A | B-cell CLL/lymphoma 11A (zinc finger protein) | 5.907613 | 6.186256 | 3.192585 | 3.091579 |
| **223196_s_at** | SESN2 | sestrin 2 | 3.284857 | 2.939274 | 8.836752 | 8.805728 |
| **223217_s_at** | NFKBIZ | nuclear factor of kappa light polypeptide gene enhancer in B-cells inhibitor, zeta | 2.458356 | 9.642678 | 9.16728 | 8.669809 |
| **223218_s_at** | NFKBIZ | nuclear factor of kappa light polypeptide gene enhancer in B-cells inhibitor, zeta | 3.053755 | 11.84149 | 11.01279 | 10.3711 |
| **223282_at** | TSHZ1 | teashirt zinc finger homeobox 1 | 5.423368 | 4.9479 | 2.756022 | 2.40992 |
| **223333_s_at** | ANGPTL4 | angiopoietin-like 4 | 3.149572 | 2.599325 | 8.352171 | 9.623024 |
| **223412_at** | KBTBD7 | kelch repeat and BTB (POZ) domain containing 7 | 7.158275 | 5.459881 | 3.415996 | 2.880465 |
| **223733_s_at** | PPP4R1L | protein phosphatase 4, regulatory subunit 1-like | 3.578179 | 3.583197 | 6.670685 | 7.194583 |
| **224336_s_at** | DUSP16 | dual specificity phosphatase 16 | 2.664368 | 2.70408 | 5.83575 | 4.908161 |
| **224489_at** | KIAA1267 | KIAA1267 | 2.933758 | 3.013631 | 6.158298 | 6.850464 |
| **225066_at** | PPP2R2D | protein phosphatase 2, regulatory subunit B, delta isoform | 3.044935 | 3.587919 | 7.670294 | 8.402227 |
| **225557_at** | CSRNP1 | cysteine-serine-rich nuclear protein 1 | 4.608158 | 6.561788 | 11.36327 | 10.92458 |
| **225671_at** | SPNS2 | spinster homolog 2 (Drosophila) | 2.28636 | 2.286237 | 2.45438 | 4.939532 |
| **226028_at** | ROBO4 | roundabout homolog 4, magic roundabout (Drosophila) | 3.211307 | 3.122909 | 7.284996 | 8.039981 |
| **226333_at** | IL6R | interleukin 6 receptor | 5.156321 | 4.606227 | 9.870235 | 10.26147 |
| **226425_at** | CLIP4 | CAP-GLY domain containing linker protein family, member 4 | 2.281685 | 2.281685 | 2.685391 | 4.557576 |
| **226560_at** |  |  | 4.504767 | 4.375418 | 8.891346 | 9.128923 |
| **227062_at** | NEAT1 | nuclear paraspeckle assembly transcript 1 (non-protein coding) | 2.40842 | 5.923897 | 8.633085 | 9.85771 |
| **227458_at** |  |  | 2.414217 | 2.545563 | 4.075569 | 6.652754 |
| **227486_at** | NT5E | 5'-nucleotidase, ecto (CD73) | 3.444171 | 2.646557 | 5.737758 | 7.79659 |
| **227803_at** | ENPP5 | ectonucleotide pyrophosphatase/phosphodiesterase 5 (putative function) | 5.734355 | 5.761135 | 3.311389 | 2.694614 |
| **227866_at** |  |  | 3.45191 | 3.989769 | 6.988041 | 7.470206 |
| **227963_at** |  |  | 4.307996 | 4.290737 | 8.871037 | 9.192254 |
| **228499_at** | PFKFB4 | 6-phosphofructo-2-kinase/fructose-2,6-biphosphatase 4 | 3.949536 | 3.54999 | 9.691752 | 10.42667 |
| **228523_at** | NANOS1 | nanos homolog 1 (Drosophila) | 7.897261 | 7.408459 | 4.079235 | 2.96103 |
| **228914_at** |  |  | 3.319558 | 4.092515 | 7.529468 | 7.459325 |
| **228923_at** | S100A6 | S100 calcium binding protein A6 | 2.772995 | 7.488204 | 8.406092 | 8.124549 |
| **229228_at** | CREB5 | cAMP responsive element binding protein 5 | 2.281685 | 2.281685 | 4.673967 | 6.060916 |
| **229318_at** |  |  | 2.301257 | 2.615906 | 4.856001 | 5.925134 |
| **229371_at** |  |  | 2.903096 | 3.572482 | 6.716189 | 6.936155 |
| **229483_at** |  |  | 2.448749 | 2.642274 | 7.698491 | 8.036509 |
| **229879_at** |  |  | 3.397458 | 3.414183 | 6.865348 | 8.370121 |
| **229927_at** | LEMD1 | LEM domain containing 1 | 2.963932 | 3.334279 | 7.631653 | 8.10541 |
| **230127_at** |  |  | 2.852493 | 3.72301 | 7.684555 | 7.495093 |
| **230183_at** | EXT1 | exostoses (multiple) 1 | 2.959717 | 2.493524 | 6.006227 | 7.212522 |
| **230345_at** | SEMA7A | semaphorin 7A, GPI membrane anchor (John Milton Hagen blood group) | 4.998873 | 3.918908 | 8.424356 | 9.147715 |
| **230372_at** | HAS2 | hyaluronan synthase 2 | 6.462691 | 6.18702 | 3.585437 | 2.646224 |
| **230683_at** | ANKRD60 | ankyrin repeat domain 60 | 2.485054 | 4.118718 | 6.815044 | 7.303942 |
| **230973_at** | SH2D5 | SH2 domain containing 5 | 3.301788 | 3.371496 | 5.968823 | 6.799853 |
| **231067_s_at** |  |  | 3.788326 | 2.482936 | 7.247718 | 7.818963 |
| **231403_at** | TRIO | triple functional domain (PTPRF interacting) | 3.38165 | 3.710175 | 7.066818 | 7.781366 |
| **232094_at** | C15orf29 | chromosome 15 open reading frame 29 | 2.332906 | 3.467909 | 5.440512 | 6.595857 |
| **232120_at** |  |  | 2.637925 | 2.726925 | 6.463455 | 5.664339 |
| **232262_at** | PIGL | phosphatidylinositol glycan anchor biosynthesis, class L | 2.281685 | 2.674314 | 4.721804 | 5.046307 |
| **232291_at** | MIR17HG | MIR17 host gene (non-protein coding) | 3.603554 | 6.661089 | 3.669092 | 3.010769 |
| **232593_at** | NEURL3 | neuralized homolog 3 (Drosophila) pseudogene | 2.890622 | 3.866727 | 5.227784 | 7.446529 |
| **232874_at** | DOCK9 | dedicator of cytokinesis 9 | 3.051341 | 3.646269 | 7.763285 | 8.550018 |
| **232918_at** | LOC541471 | hypothetical LOC541471 | 2.965693 | 3.022483 | 5.308385 | 5.948857 |
| **232925_at** |  |  | 3.927087 | 4.45462 | 8.345465 | 7.456189 |
| **233044_at** |  |  | 2.870355 | 2.9904 | 7.115439 | 4.63897 |
| **233303_at** |  |  | 3.108782 | 6.460546 | 4.333355 | 2.655183 |
| **233691_at** |  |  | 2.281685 | 2.354543 | 4.607718 | 4.37395 |
| **233771_at** |  |  | 2.562958 | 2.562958 | 5.322132 | 3.568015 |
| **233921_s_at** |  |  | 3.561933 | 5.420347 | 9.344239 | 7.584109 |
| **234269_at** |  |  | 2.281685 | 2.281685 | 4.436349 | 2.314021 |
| **234608_at** | LAMA3 | laminin, alpha 3 | 2.913481 | 3.987875 | 7.145692 | 5.226024 |
| **235008_at** |  |  | 3.344576 | 4.453506 | 8.171265 | 7.996211 |
| **235086_at** | THBS1 | thrombospondin 1 | 2.355666 | 2.608489 | 5.23778 | 5.914431 |
| **235102_x_at** |  |  | 2.985477 | 6.446449 | 8.118193 | 7.794996 |
| **235419_at** |  |  | 2.925624 | 5.283659 | 9.807212 | 10.25054 |
| **235421_at** | MAP3K8 | mitogen-activated protein kinase kinase kinase 8 | 3.009027 | 6.032157 | 2.688393 | 2.867717 |
| **235490_at** | TMEM107 | transmembrane protein 107 | 2.81908 | 4.871221 | 8.121446 | 7.66732 |
| **235646_at** |  |  | 2.478389 | 4.224701 | 6.388576 | 6.357212 |
| **235693_at** |  |  | 5.400177 | 4.671864 | 2.516541 | 2.411192 |
| **235705_at** |  |  | 3.520543 | 5.382811 | 8.132958 | 8.644692 |
| **235845_at** | SP5 | Sp5 transcription factor | 8.361489 | 7.954128 | 4.915609 | 3.384086 |
| **236215_at** |  |  | 2.766025 | 5.232363 | 2.554231 | 2.283091 |
| **236423_at** |  |  | 2.406107 | 2.497394 | 3.659353 | 5.005305 |
| **236474_at** |  |  | 2.570016 | 2.570076 | 5.2781 | 3.186077 |
| **236610_at** |  |  | 9.451811 | 7.914748 | 5.966338 | 3.505208 |
| **236781_at** |  |  | 2.281685 | 2.281958 | 2.975063 | 5.538937 |
| **236889_at** |  |  | 2.281685 | 4.633312 | 6.251229 | 6.123442 |
| **237238_at** | WWC1 | WW and C2 domain containing 1 | 2.328293 | 2.38323 | 5.40309 | 6.008207 |
| **237249_at** | KCNQ1OT1 | KCNQ1 overlapping transcript 1 (non-protein coding) | 2.85427 | 3.509461 | 4.457984 | 6.808633 |
| **237361_at** |  |  | 2.583477 | 2.481613 | 5.879305 | 6.700129 |
| **237435_at** |  |  | 2.755212 | 2.439997 | 4.585578 | 6.018835 |
| **237737_at** | LOC727770 | similar to FLJ00310 protein | 2.282547 | 2.282547 | 2.504983 | 5.538643 |
| **238567_at** | SGPP2 | sphingosine-1-phosphate phosphotase 2 | 3.450433 | 2.825542 | 6.974797 | 7.118174 |
| **238716_at** |  |  | 2.281685 | 2.595565 | 3.123706 | 5.51186 |
| **238827_at** |  |  | 3.720339 | 4.405558 | 8.09667 | 8.640919 |
| **238988_at** |  |  | 2.428612 | 4.447476 | 6.649223 | 5.874866 |
| **239157_at** | ZSCAN12L1 | zinc finger and SCAN domain containing 12-like 1 | 2.369806 | 2.367238 | 3.957486 | 5.721113 |
| **239203_at** | C7orf53 | chromosome 7 open reading frame 53 | 3.762495 | 3.762495 | 6.817211 | 8.248527 |
| **239227_at** |  |  | 2.968491 | 3.359884 | 5.070185 | 6.657901 |
| **239251_at** |  |  | 2.824705 | 4.198964 | 7.910281 | 7.528128 |
| **239264_at** |  |  | 2.916249 | 5.629815 | 2.916249 | 2.34033 |
| **239430_at** | IGFL1 | IGF-like family member 1 | 2.553931 | 2.798212 | 7.230663 | 7.940164 |
| **239439_at** | AFF4 | AF4/FMR2 family, member 4 | 2.654615 | 2.621326 | 4.969944 | 5.163605 |
| **239516_at** |  |  | 5.399396 | 6.364431 | 3.291299 | 2.996525 |
| **239652_at** |  |  | 2.339275 | 2.339275 | 5.708023 | 6.794878 |
| **239721_at** |  |  | 3.114685 | 4.17349 | 7.484622 | 7.257722 |
| **239751_at** |  |  | 2.31757 | 2.317711 | 6.338905 | 7.077768 |
| **239756_at** |  |  | 3.428158 | 3.402487 | 6.776903 | 7.813965 |
| **239876_at** |  |  | 2.606502 | 6.445415 | 2.609044 | 2.609044 |
| **240297_at** |  |  | 5.06624 | 3.207028 | 2.281685 | 2.368549 |
| **240502_at** |  |  | 2.404901 | 4.373062 | 6.672605 | 6.067263 |
| **240991_at** |  |  | 3.548482 | 2.480675 | 8.254445 | 8.300982 |
| **241001_at** |  |  | 2.281685 | 2.309846 | 5.907205 | 6.200932 |
| **241722_x_at** |  |  | 2.409735 | 7.075499 | 6.297254 | 2.409735 |
| **241824_at** |  |  | 2.429329 | 4.817031 | 6.922539 | 6.351799 |
| **242310_at** |  |  | 2.320145 | 2.483846 | 2.852102 | 4.72871 |
| **242329_at** |  |  | 4.016866 | 2.961096 | 6.300186 | 8.630524 |
| **242556_at** |  |  | 2.633646 | 4.162645 | 8.020356 | 6.906529 |
| **242727_at** | ARL5B | ADP-ribosylation factor-like 5B | 3.858919 | 4.108356 | 7.802782 | 8.488255 |
| **242814_at** | SERPINB9 | serpin peptidase inhibitor, clade B (ovalbumin), member 9 | 2.313376 | 2.313376 | 4.275829 | 5.523802 |
| **242963_at** | SGMS2 | sphingomyelin synthase 2 | 2.59215 | 2.950426 | 7.128067 | 7.786397 |
| **243003_at** |  |  | 2.458647 | 5.036483 | 2.432765 | 2.459982 |
| **243031_at** |  |  | 2.894837 | 2.894837 | 6.249347 | 4.881944 |
| **243296_at** | NAMPT | nicotinamide phosphoribosyltransferase | 3.284148 | 8.895185 | 8.178758 | 7.90942 |
| **244023_at** | SYK | spleen tyrosine kinase | 7.347731 | 8.737587 | 3.789065 | 2.755334 |
| **244292_at** |  |  | 2.827916 | 5.348718 | 7.71801 | 7.379389 |
| **244322_at** |  |  | 2.567854 | 2.90384 | 7.617333 | 8.367269 |
| **244387_at** |  |  | 3.72954 | 6.087994 | 2.711284 | 2.775002 |
| **244427_at** | KIF23 | kinesin family member 23 | 3.199198 | 6.012029 | 2.41351 | 4.002943 |
| **244447_at** |  |  | 2.908918 | 8.77146 | 7.606937 | 6.606711 |
| **244524_at** |  |  | 2.301917 | 2.417227 | 7.093955 | 7.178235 |
| **244548_at** |  |  | 3.272881 | 3.434446 | 5.872949 | 7.523027 |
| **36829_at** | PER1 | period homolog 1 (Drosophila) | 3.344138 | 3.42975 | 7.412292 | 8.333033 |
| **38037_at** | HBEGF | heparin-binding EGF-like growth factor | 2.609918 | 2.61037 | 8.764212 | 9.887068 |
| **39248_at** | AQP3 | aquaporin 3 (Gill blood group) | 3.263078 | 2.90888 | 6.944878 | 7.868351 |
| **40016_g_at** | MAST4 | microtubule associated serine/threonine kinase family member 4 | 2.802087 | 2.727112 | 6.034087 | 6.97583 |
| **41386_i_at** | KDM6B | lysine (K)-specific demethylase 6B | 3.702807 | 6.310406 | 9.789451 | 9.189477 |
| **41387_r_at** | KDM6B | lysine (K)-specific demethylase 6B | 3.209465 | 4.202714 | 8.6084 | 7.835326 |
