# Supplementary material for: Phenotypic Pattern-Based Assay for Dynamically Monitoring Host Cellular Responses to Salmonella Infections
Source: PLoS One. 2011 Nov 3;6(11):e26544. doi: 10.1371/journal.pone.0026544 (PMC3207827; doi:10.1371/journal.pone.0026544)
Supplement: Table S2 — Gene ontology terms most highly represented in genes with significantly different expression. (DOC) [file pone.0026544.s006.doc]

**Table S2. Gene ontology terms most highly represented in genes with significantly different expression**

| **GO term IDs** | **Enriched Go terms** | **Count** | **Total** | **P-value** |
| --- | --- | --- | --- | --- |
| GO:0007154 | cell communication | 40 | 1698 | 5.54E-07 |
| GO:0009605 | response to external stimulus | 14 | 239 | 2.02E-05 |
| GO:0005102 | receptor binding | 16 | 323 | 2.02E-05 |
| GO:0005125 | cytokine activity | 9 | 81 | 2.02E-05 |
| GO:0007165 | signal transduction | 34 | 1544 | 4.47E-05 |
| GO:0009611 | response to wounding | 11 | 158 | 4.47E-05 |
| GO:0042330 | taxis | 7 | 48 | 4.47E-05 |
| GO:0006935 | chemotaxis | 7 | 48 | 4.47E-05 |
| GO:0005615 | extracellular space | 11 | 160 | 4.49E-05 |
| GO:0007267 | cell-cell signaling | 13 | 239 | 4.95E-05 |
| GO:0008083 | growth factor activity | 8 | 74 | 4.95E-05 |
| GO:0007626 | locomotory behavior; | 7 | 62 | 0.000178 |
| GO:0007166 | cell surface receptor linked signal transduction | 16 | 526 | 0.000195 |
| GO:0032501 | multicellular organismal process | 29 | 1321 | 0.000211 |
| GO:0044421 | extracellular region part | 12 | 244 | 0.000275 |
| GO:0042379 | chemokine receptor binding | 4 | 13 | 0.000328 |
| GO:0008009 | chemokine activity; | 4 | 13 | 0.000328 |
| GO:0007610 | behavior | 8 | 102 | 0.000339 |
| GO:0002521 | leukocyte differentiation | 6 | 48 | 0.000339 |
| GO:0006952 | defense response | 10 | 180 | 0.000477 |
| GO:0030098 | lymphocyte differentiation | 5 | 34 | 0.000819 |
| GO:0030183 | B cell differentiation | 4 | 17 | 0.000819 |
| GO:0048513 | organ development | 15 | 525 | 0.00089 |

Total number of candidate genes= 272, total number of reference genes= 27,359. Gene Ontology (GO) term IDs and corresponding GO terms are shown, “Count”= number of candidate genes with that annotation, “Total”= total number of reference genes with that annotation^1^.
