# Supplementary material for: Phenotypic Pattern-Based Assay for Dynamically Monitoring Host Cellular Responses to Salmonella Infections
Source: PLoS One. 2011 Nov 3;6(11):e26544. doi: 10.1371/journal.pone.0026544 (PMC3207827; doi:10.1371/journal.pone.0026544)
Supplement: Table S3 — Pathways in response of HT29 epithelia to S. typhimurium . (DOC) [file pone.0026544.s007.doc]

**Table S3. Pathways in response of HT29 epithelia to *S. typhimurium***

| **pathways** | **Total gene** | **P-value** | **Gene** |
| --- | --- | --- | --- |
| Jak-STAT signaling pathway | 8 | 0 | CLCF1,IL24,PIK3CD,IL11,IL10RA,CSF3,IL6R,CSF2 |
| Cytokine-cytokine receptor interaction | 17 | 0 | CLCF1,IL24,TNFRSF10B,TNFRSF25,VEGFA,  CCL20,TNFRSF9,IL11,IL10RA,IL8,IL6R,CXCL2,  CXCL3,CXCL1,CSF3,CSF2,CCR7 |
| MAPK signaling pathway | 9 | 10E-6 | DUSP16,DUSP10,JUN,NR4A1,FLNC,FGFR1,  DUSP8,DUSP5,MAP3K8 |
| ErbB signaling pathway | 5 | 1.4E-5 | PIK3CD,JUN,HBEGF,AREG,ABL2 |
| Focal adhesion | 6 | 8.9E-5 | VEGFA,THBS1,PIK3CD,JUN,LAMA3,FLNC |
| p53 signaling pathway | 4 | 1.04E-4 | SESN2,TNFRSF10B,THBS1,SERPINE1 |
| Epithelial cell signaling in Helicobacter pylori infection | 4 | 1.1E-4 | JUN,IL8,CXCL1,HBEGF |
| Natural killer cell mediated cytotoxicity | 5 | 1.3E-4 | TNFRSF10B,SYK,PIK3CD,GZMB,CSF2 |
| Hematopoietic cell lineage | 4 | 2.55E-4 | IL11,IL6R,CSF3,CSF2 |
| Bladder cancer | 3 | 4.34E-4 | VEGFA,THBS1,IL8 |
| Toll-like receptor signaling pathway | 4 | 4.67E-4 | PIK3CD,JUN.IL8.MAP3K8 |
| T cell receptor signaling pathway | 4 | 6.0E-4 | PIK3CD,JUN,MAP3K8,CSF2 |
| Circadian rhythm | 2 | 9.22E-4 | BHLHE40,PER1 |

Significant pathways with P-value less than 0.001 using KEGG pathway resources were listed.
