# Supplementary material for: Phenotypic Pattern-Based Assay for Dynamically Monitoring Host Cellular Responses to Salmonella Infections
Source: PLoS One. 2011 Nov 3;6(11):e26544. doi: 10.1371/journal.pone.0026544 (PMC3207827; doi:10.1371/journal.pone.0026544)
Supplement: Table S4 — MICs of eight antibiotics for Salmonella strain SL1344. (DOC) [file pone.0026544.s008.doc]

**Table S4. MICs of eight antibiotics for *Salmonella* strain SL1344**

| Antibiotic | MIC (mg/L) |
| --- | --- |
|  | *S. typhimurium* |
| Ampicillin | 1 |
| Chloramphenicol | 2 |
| Gentamycin | 2 |
| Kanamycin | 4 |
| Levofloxacin | 0.125 |
| Penicillin | 8 |
| Streptomycin | >128 |
| Tetracycline | 2 |
