# Supplementary material for: Phenotypic Pattern-Based Assay for Dynamically Monitoring Host Cellular Responses to Salmonella Infections
Source: PLoS One. 2011 Nov 3;6(11):e26544. doi: 10.1371/journal.pone.0026544 (PMC3207827; doi:10.1371/journal.pone.0026544)
Supplement: Supplemental Methods S1 — (DOC) [file pone.0026544.s009.doc]

**Supplemental Methods S1**

**Preparation of *Salmonella* conditioned medium.** *S. typhimurium* was grown overnight in 5 ml of LB broth without agitation at 37°C, diluted 1:50 in LB broth and grown approximately 6 h without agitation at 37°C. Bacteria were harvested by centrifugation at 5,000 x *g* for 3 min and washed with PBS and finally resuspended in RPMI medium 1640 without fetal bovine serum at a density of 2×10^7^ cfu/ml. Culture was incubated at 37°C and 5% CO_2_ for 6 h and centrifuged at 13,000 x *g* for 3 min. Supernatant (conditioned medium) was filtered through a 0.22 μm filter (Millipore) to remove all bacteria cells and stored at -80°C before further experiment.

**Cell Proliferation and Viability Assays**

HT-29 cells were infected with *S. typhimurium* SL1344 at MOI = 200. At 0, 0.75, 1.5, 3, 5, and 7h post-infection, surviving, adherent cells were fixed with 4% formaldehyde and stained with 5'-Bromo-2'-deoxy-uridine (BrdU), phosphor-histone, and DAPI (Thermo Scientific).

**DNA fragmentation assay**

DNA was isolated with Apoptotic DNA Ladder Kit (Beyotime). Samples (~ 3 ug DNA per lane) were separated by electrophoresis on 1.5% agarose gel and visualized by ethidium bromide staining.

**Scanning electron microscopy**

HT-29 cells grown on glass coverslip were washed several times with PBS and fixed in cold (4°C) 2% gluteraldehyde, 0.1 M sodium phosphate buffer (pH 7.4) overnight. After washing with phosphate buffer, samples were postfixed in cold 1% OsO_4_ in 0.1 M phosphate buffer for 90 min, and then stained with cold 0.25% uranyl acetate overnight. Samples were dehydrated in a critical point apparatus and, after a gold evaporation step, were examined with a Leica Stereoscan 260 scanning electron microscopy.

**RNA isolation and microarray experiment.**

One uninfected sample (0h) and three infected cell samples were collected at 45 min, 3 hour and 7 hour post-infection for microarray analysis. The isolation of total RNA with TRIzol (Invitrogen) was performed according to the manufacturer’s recommendation. The purity and yield of RNA were determined by the ratio of OD260/280 and OD260 measured by Nanodrop (Thermo). RNA integrity was examined by Agilent 2100 bioanalyzer. An aliquot of 2 μg of total RNA was used for reverse transcription. After double stranded cDNA synthesis, MessageAmpTM Ⅱ aRNA Amplification Kit was used to produce biotin-tagged cRNA. The resulting bio-tagged cRNA were fragmented to strands of 35-200 bases in length. The fragmented cRNA was hybridized to Affymetrix Human Genome U133 Plus 2.0 Array. Hybridization was performed at 45°C with rotation for 16 hours. The arrays were washed and stained with streptavidin phycoerythrin on an Affymetrix Fluidics Station 450 followed by scanning on a GeneChip Scanner.

**Microarray Data Analysis.** The scanned images were extracted then analyzed to generate raw data files saved as CEL files using GeneChip Operating software (GCOS 1.4). R software (version 2.10.0) and bioconductor packages ([www.bioconductor.org](http://www.bioconductor.org/)) were using for array data analysis. GCRMA method was performed to normalize the different arrays. After removing control probes and absent probes, a coefficient variation (CV) curve was used to find differentially expressed genes. Probesets with CV values greater than 99% percentile of the distribution corresponding to CV were identified as significantly differentially expressed genes during infection. In order to further elucidate the functions of significantly differentially expressed genes during infection, we examined gene ontologies using a web-based analysis tool GOstat (<http://gostat.wehi.edu.au/>). The significantly differentially expressed genes were compared genes which were present at any array as a reference. The GO terms were ranked according their P-value. Differentially expressed probesets were analyzed using a free web-based Molecular Annotation System (MAS, version 2.0, [www.capitalbio.com](http://www.capitalbio.com/)). After analysis, the pathways are ranked with statistical significant by calculating their P-value based on hyper-geometric distribution.

**MICs determination**

A broth dilution method was carried out to determine the MICs of ampicillin, chloramphenicol, gentamycin, kanamycin, levofloxacin, penicillin, and streptomycin [2]. Antibiotics were prepared and diluted according to their manufacturers’ recommendations. Overnight cultures of *Salmonella* were diluted into Mueller Hinton (MH) broth, and aliquoted into 96-well microliter plates that contained increasing concentration of the antibiotics (0 to 256 mg/L). the final inoculum contained approximately 5×10^5^ CFU/ml. The absorbance of the cultures (at 600 nm) was measured after 20 h of incubation at 37°C without shaking.

**Intracellular activity of antibiotics**

*Salmonella* were grown overnight in 5 ml of LB broth without agitation at 37°C, diluted 1:50 in fresh LB broth and cultured approximately 6 h without agitation at 37°C before use. *Salmonella* were harvested by centrifugation and washed with PBS and finally suspended in DMEM. Before experimental use, the J774A.1 cells were washed with PBS twice and starved in DMEM for 3 h. After starvation step, J774A.1 cells were infected with *Salmonella* at an MOI of 200 for 1 h to allow the bacteria to invade cells. Infected cells were washed twice to remove extracellular bacteria. Warmed DMEM containing 100 μg/ml gentamycin was added to the flask for 30 min to kill the remaining free or bound extracellula bacteria. The infected cells were washed with PBS twice, scraped off with a cell scraper (Corning) and seed into E-plate containing antibiotics. The CI values were measured every 5 min.
